# Latent subdimensions of anxiety and depression differentially influence exertion of effort in pursuit of reward versus avoidance of threat

**DOI:** 10.1038/s41398-026-04194-0

**Published:** 2026-06-30

**Authors:** Jennifer D. Senta, Anne G. E. Collins, Peter Dayan, Sonia J. Bishop

**Affiliations:** 1 Helen Wills Neuroscience Institute, University of California, Berkeley, CA USA; 2 Department of Psychology, University of California, Berkeley, CA USA; 3 Max Planck Institute of Biological Cybernetics, Tübingen, Germany; 4 University of Tübingen, Tübingen, Germany; 5 School of Psychology, Trinity College Dublin, Dublin, Ireland; 6 Trinity College Institute of Neuroscience, Trinity College Dublin, Dublin, Ireland

**Keywords:** Human behaviour, Depression

## Abstract

Anxiety and depression are highly comorbid. Across species, depression-related phenotypes have been linked to reduced willingness to expend effort to pursue rewards. Effortful threat avoidance has been less extensively studied. Here, we used a dimensional model of psychopathology to investigate whether distinct sub-dimensions of depressed and anxious affect differentially impact effortful reward pursuit versus threat avoidance in humans. Hierarchical Bayesian modeling revealed that component decision-making mechanisms underlying effortful reward pursuit were differentially impacted by latent dimensions capturing the depressive symptoms of apathy versus anhedonia. Meanwhile, only the latent dimension tapping worry-related symptoms significantly impacted effortful threat avoidance, increasing willingness to exert effort to avoid threat and reducing sensitivity to threat magnitude.

## Introduction

Everyday life is characterized by decisions in which individuals must evaluate the costs versus benefits of various actions. One frequently encountered form of cost comes from the exertion of physical effort. This might be required to attain a positive reward or to avoid a negative outcome. For example, someone might ask themselves if the effort required to go to a party is worth the reward of enjoying it, or whether avoiding a dangerous neighborhood is worth walking some distance out of their way. Individuals vary in how much physical effort they are willing to exert in pursuit or avoidance of specific outcomes, and aberrant willingness to exert effort has been associated with a variety of psychopathological conditions [1–5].

A large body of work in rodents has established a relationship between depressive phenotypes and reduced willingness to exert effort in pursuit of reward. Effortful reward pursuit in such paradigms typically entails physical lever presses to gain a desirable food and involves choice selection between a low effort/low reward option and a high effort/high reward option [6, 7]. Findings link depressive phenotypes to low levels of high effort/high reward choice selection [8–12]; with this behavior being normalized by the administration of antidepressant agents [8, 9].

In humans, effortful reward pursuit is usually studied with rewards consisting of monetary gain [2, 4, 13–15]. A human analog of the rodent task described above, the Effort Expenditure for Rewards Task (EEfRT) [15], which pits a low effort/low reward option against a high effort/high reward option, has been used in studies of patients with depressive disorders. Findings from these studies have been broadly consistent with the animal literature, with patients with major depressive disorder (MDD) showing lower selection of high effort/high reward options than healthy control participants [16–19]. Among medicated MDD patients in remission from depression, reduced high effort/high reward choice selection has also been shown to be predictive of future depressive relapse [20].

In addition to the use of binary psychiatric diagnoses such as MDD, researchers have used well-validated standardized questionnaires to examine the relationship between continuous measures of depressive symptomatology and effortful reward pursuit. Elevated levels of both apathy and anhedonia have been associated with reduced effortful reward pursuit [4, 14, 17, 21, 22]. However, it remains unclear whether these dimensions of depressive symptomatology influence effortful reward pursuit via common or distinct mechanisms. In an important step towards addressing this, Bonnelle and colleagues devised a task that orthogonally manipulated the levels of required effort and levels of possible reward (referred to as ‘stake’) that might be obtained, enabling individual differences in effort cost valuation versus reward sensitivity to be teased apart. The authors focused on behavioral apathy, finding it to be linked to increased subjective effort costs at the lowest levels of reward in one study [22] and elevated sensitivity to increases in the level of required effort in a second [21].

Scores on self-report measures of different aspects of depressive symptomatology, such as anhedonia and apathy, tend to be highly correlated with each other [23], while scores on measures of depression are themselves highly correlated with scores on self-report measures of anxiety [24, 25]. This raises the question of whether the findings reported to date are specific to anhedonia or apathy, linked to a broader depressive phenotype, or even common to both depression and anxiety. Husain and colleagues controlled for anhedonia and depressive symptoms in their apathy analyses but did not fully report findings for these other symptom dimensions. Further, despite the high comorbidity between anxiety and depression [26–28], there has been little human research investigating the relationship of anxiety with individual differences in effortful reward pursuit. This contrasts with the widespread use of instrumental avoidance paradigms in human anxiety research (e.g., [29, 30]; see [31] for a review). These studies build on the extensive basic neuroscience literature on the instrumental avoidance of threat [32–35]. Here, however, effort costs are typically not manipulated. As we have hypothesized in prior theoretical work [1], if anxiety is associated with *increased* willingness to exert effort to avoid threat whereas depression is associated with *reduced* willingness to exert effort to obtain reward this might potentially account, at least in part, for the differential patterns of behavior linked to anxiety and depression. We aimed to address this in the study reported here.

Specifically, we sought to characterize fully the relationship between distinct components of anxious and depressive affect and alterations, or biases, in the computations underlying effort-based decision-making. To achieve this, we used bifactor modeling of item-level questionnaire responses, together with a hierarchical Bayesian modeling framework, to examine the influences of latent subdimensions of depression and anxiety upon effortful pursuit of reward and avoidance of punishment. The reward version of the effort-based decision-making (EBDM) task used was closely based on the task developed by Bonnelle and colleagues to facilitate dissociation of subjective valuation of required effort from subjective valuation of potential reward. To investigate participants’ willingness to expend effort to avoid aversive outcomes, we created a structurally equivalent task using electrical stimulation of different levels as the aversive outcome. Our specific aims were as follows: First, we sought to investigate whether distinct latent subdimensions of depression impact the same or different component mechanisms of decision-making involved in effortful reward pursuit. Second, given the more modest literature on the influence of anxiety on effortful decision-making [1], we sought to determine whether cognitive or physiological symptoms of anxiety are associated with differences in effortful decision-making, and if so, whether this varies between effortful reward pursuit and effortful threat avoidance. Finally, we sought to determine whether effortful decision-making in either, or both, pursuit of reward and avoidance of threat differentiates individuals with elevated anxious versus depressive symptomatology.

## Methods

### Modeling latent dimensions of negative affect

#### Participants

We used two distinct datasets collected through Qualtrics online for exploratory (n = 453) and confirmatory (n = 252) factor analyses. The winning bifactor model was then applied to item responses from participants who completed the in-person effort-based decision-making study (described below).

The first online dataset, used for exploratory bifactor analysis, comprised data from n = 285 participants recruited from UC Berkeley’s research participation pool (RPP) and data from n = 168 participants recruited via Amazon Mechanical Turk (AMT); n = 453 total, comprising 189 male and 264 female participants, age range 18-45 years (exact age not collected). Participants recruited through AMT were compensated $12. Participants through RPP were compensated with 1.5 psychology course credits. Each participant completed online informed consent prior to completion of the questionnaire battery. All methods were performed in accordance with the relevant guidelines and regulations and approved by the UC Berkeley Committee for the Protection of Human Subjects under protocol 2023-03-16131.

The second online dataset, used for confirmatory analysis, included n = 252 participants recruited via AMT (comprising 97 male and 155 female participants, average age 42.8 + /- 12.1 years) who participated in a separate online behavioral study conducted by our lab. This online behavioral study included the questionnaire battery used in the current study. These participants also completed an online behavioral learning task; however, only the relevant questionnaire data were used in the current study. Each participant completed online informed consent prior to participation in this study. All methods were performed in accordance with the relevant guidelines and regulations and approved by the UC Berkeley Committee for the Protection of Human Subjects under protocol 2023-03-16131. Participants were compensated $20 for participating in the study, plus a $5 bonus to incentivize performance during the behavioral task (not analyzed here).

#### Exclusion criteria

Upon session completion, all participants recruited through AMT were directed to a final screen in which they were informed that they had completed the session and that their payment was guaranteed, and they were asked to respond honestly to three final questions to aid in the accuracy of our research (following [36]). Participants were asked to confirm 1) whether they were physically located in the United States, 2) whether they had used cannabis in the previous two weeks, and 3) whether they thought we should include their data in our study based on their own judgment of their attentiveness and honesty. Responses to these questions indicating lack of attention, cannabis use or location outside the United States were used to exclude participants from later analysis. In addition, we embedded questionnaire attention checks throughout the online questionnaires for AMT participants (e.g., ‘Select ‘b’ to show you are paying attention’); participants who failed these checks were also excluded. A total of n = 86 participants were excluded from the EFA data and n = 29 from the CFA data (resulting in the final post-exclusion counts reported above of n = 453 for the EFA data set and n = 252 for the CFA data set).

#### Questionnaire measures

Scales were selected to span a range of internalizing negative affect symptoms. We augmented a questionnaire battery used in previous research [37] to include measures of apathy and anhedonia. The measures used were as follows: the Beck Depression Inventory [38] (BDI); the Penn State Worry Questionnaire [39](PSWQ); the Mood and Anxiety Symptom Questionnaire [25](MASQ) anhedonic depression (AD) and anxious arousal (AA) subscales; the State Trait Anxiety Inventory [40] trait (STAI-Trait) questionnaire (which itself comprises both depressed and anxious subscales [41]); the Apathy and Motivation Index [23] (AMI) behavioral (-b) subscale; and the Temporal Experience of Pleasure [42] (TEPS) anticipatory hedonia (-ant) subscale. Items evaluating suicidality were removed for online administration; total number of response items = 110. See Table S1 for participant subscale scores.

#### Bifactor modeling of questionnaire item-level responses

Bifactor modeling of item-level responses was used to parse variance linked to a higher-order general factor from that linked to lower-level specific factors. This approach has shown considerable success in capturing symptom variance associated with psychopathological conditions such as depression and anxiety, which are characterized by both shared and unique symptoms [37, 43–45] (note, see [46] for an alternative perspective and [47] for rebuttal). To date, there has been limited use of bifactor modeling of internalizing symptoms within the field of computational psychiatry.

To account for the ordinal nature of the item responses, polychoric correlations were used in all analyses. Sampling adequacy was confirmed using the Kaiser-Meyer-Olkin (KMO) test, which yielded a sampling adequacy of 0.82 (values over 0.80 are considered adequate) on the polychoric item correlation matrix for the exploratory dataset. A parallel eigenvalue decomposition analysis, which uses resampling techniques to compare the covariance of item responses with identically sized matrices generated from random Gaussian noise indicated that up to 21 factors might be reliably distinguished from noise. This number of identifiable factors might reflect the large sample size and the associated possibility of overfitting.

We performed exploratory bifactor analyses, specifying one general factor and varying the numbers of specific factors. Bifactor analysis was conducted in R using the psych package omega function. The Schmid-Leiman procedure [48] was used to transform an oblique factor analysis of the full item response data into a bifactor structure with orthogonality of the common and specific factors. Resulting factor loadings were thresholded to only include loadings > 0.2.

A comparison of Bayesian Information Criteria (BIC) scores, which assess model fit while penalizing for complexity, showed continued improvement in exploratory model fit as additional specific factors (up to seven, the maximum tested) were included. We next assessed the fit of each model to the confirmatory data set using both confirmatory fit index (CFI) and Tucker-Lewis measure of fit. Confirmatory fit measures of 0.95 or higher are considered a strong fit.

To check the face validity of the factors that comprised the winning bifactor model, we calculated a factor score for each participant whose data was included in the exploratory dataset using the Anderson-Rubin method, which preserves orthogonality of the general and specific scores [49]. We then assessed the correlation of these factor scores with participants’ summary scores for the original questionnaire scales and subscales.

### Effort-based decision making study

#### Participants

Participants were recruited from the UC Berkeley student research participation pool (RPP) and the local community. A joint pre-screening survey approved by the UC Berkeley Committee for the Protection of Human Subjects (CPHS) was administered to students enrolled in the UC Berkeley Research Participation Pool (RPP), with informed consent being obtained prior to RPP enrolled students completing the pre-screening. This survey included the STAI and MASQ questionnaires and was used to ensure participants showed a good range of scores across these scales. Potential participants were further screened using a medical questionnaire and excluded if they reported cannabis use within the previous 2 weeks, or antidepressant or anxiolytic medication usage. In addition, participants with a history of fainting, vasovagal syncope, or any heart condition or pacemaker-like devices were excluded. Sixty-one participants completed the behavioral experiment over two sessions in testing rooms within the Department of Psychology at UC Berkeley. Data from eleven participants were excluded from analysis: 5 due to equipment failure and 6 due to failure to fully complete both sessions. The final participant group (n = 50) comprised 19 male and 31 female participants, with average age (+/- standard deviation) of 22 + /- 2.3 years.

#### Procedure

Participants completed two in-lab sessions, each of a maximum 2 h duration. These were completed on different days to ameliorate the effects of fatigue but were restricted to be within 5 days of each other. Each participant provided written informed consent prior to participation in each study session. In each session, participants completed questionnaires and performed either the reward or aversive versions of the Effort Based Decision Making (EBDM) task (counterbalanced across sessions.) All methods were performed in accordance with the relevant guidelines and regulations and approved by the UC Berkeley Committee for the Protection of Human Subjects under protocol 2023-02-16046. Participants could choose whether to receive psychology course participation credit in the amount of 1 credit per hour as their main source of study compensation (UC Berkeley students only, credited in accordance with UC Berkeley RPP policies), or to receive cash compensation of $15 per hour for each of the two study sessions. Study sessions were scheduled and paid for a minimum of 2 h each. All participants received a $2.50 bonus during each session to incentivize maximal effort during effort calibration. Participants also received a $15 bonus upon completing the second session to incentivize completion of both sessions. In the reward task session, participants were able to earn a performance bonus of up to $7.50.

#### Questionnaires

The questionnaires were as described above. They were divided across the two sessions and administered prior to task performance. See Table S1 for participant scale and subscale scores. We used the factor loadings from the winning bifactor model to calculate factor scores for each participant using the same methodology as described above [49]. These factor scores were correlated with participants’ summary scores on the original questionnaire scales and subscales to determine if the pattern obtained was consistent with the findings for the online exploratory dataset.

#### Calibration of effort

Participants exerted effort in both versions of the task by applying squeezing pressure to a dynamometer (hand grip) device from BioPac with their dominant hand. Following task instructions, but before any study trials, the maximum level of force to be used in the experiment was calibrated to be challenging but attainable for the individual participant. To perform the calibration, participants were shown an empty tree with a top ladder rung set to a very high difficultly level (no participants reached or exceeded the initial top rung difficulty level during calibration). Participants were told to squeeze with their maximal force while remaining comfortable, and that they might receive a bonus if they climbed high enough on the tree. Participants were then given two more such trials, with the instruction to see if they could exceed their previous height. All participants received the full bonus regardless of actual effort exerted during calibration.

The maximum force applied by a given participant during calibration was set as the Maximum Voluntary Contraction (MVC) [22]. Since our task required participants to briefly sustain the required force for a period of at least 0.5 s, and since participants completed 6 blocks of trials, we set 85% of the MVC as the participant’s “sustainable” MVC (SMVC). The highest level of effort required for the task was set equal to the SMVC, and each lower level of effort was reduced by 10% of the SMVC.

Participants performed the calibration at the beginning of each session to account for possible inter-day differences in grip strength. However, the SMVC on the second session was capped such that it did not vary by more than 15% from the initial session SVMC.

#### Calibration of electrical stimulation

In the threat avoidance version of the EBDM task, electrical stimulation was delivered via a DigiTimer constant current stimulator model DS7A. Prior to the start of the session, the electrode was sanitized and attached to the inside of the wrist of the participant’s non-dominant hand using hypoallergenic medical tape. The experimenter adjusted the voltage to just above zero and administered single 2 ms test pulses while increasing the voltage until the participant reported that they could feel a sensation similar to that of a small pinprick. This level of voltage was then set as the participant’s baseline (level 1).

We next calibrated the maximum amount of stimulation that might be delivered to the participant during the task. Starting with a single level 1 pulse, we increased the number of level 1 pulses, asking the participant to rate each stimulation burst on a scale of 1 to 10, informing them that a subjective level 7 was the highest level we would ever administer. Participants were instructed that level 7 should be a level that they would wish to avoid, but which they found tolerable, and which would not cause them any lasting distress. Once a participant indicated that their level 7 had been reached, we randomly sampled 14 additional ratings by repeating various numbers of pulses between but not inclusive of levels 1 through 7 and asking the participant to rate each burst of stimulation. Previous literature has indicated a non-linear (sigmoidal) relationship between increasing numbers of electrical pulses and increased subjective ratings of stimulation aversiveness [50]. Once ratings were complete, a sigmoid curve was fit to the ratings data between levels 1 and 7 (see Figure S1). Evenly spaced points on the curve were defined to represent the intermediate stimulation levels 2-6. In the rare case that a participant responded unreliably to the varying pulse numbers and a sigmoid curve could not be fit, the calibration procedure was repeated. Calibration was also repeated if a participant reached their subjective level 7 before 8 pulses had been delivered or did not reach their subjective level 7 by the time 32 pulses were delivered. Once the sigmoid curve had been successfully fit to the participant’s final calibration data, the subjective ratings and corresponding number of pulses were used to determine the stimulation delivered in the threat avoidance version of the EBDM task (described below).

#### The effort-based decision-making task: reward pursuit version

After instructions, calibration of force, and two practice trials, each participant completed 6 blocks of 26 trials each, with a 10-s rest period after every 5 trials, and a 30-s rest period after each block. Trials were designed such that each block contained one full set of orthogonally manipulated combinations of the 5 possible effort levels and 5 possible reward levels, plus one additional trial per block. Trials were pseudo-randomized and fixed across participants.

**Fig. 1:**
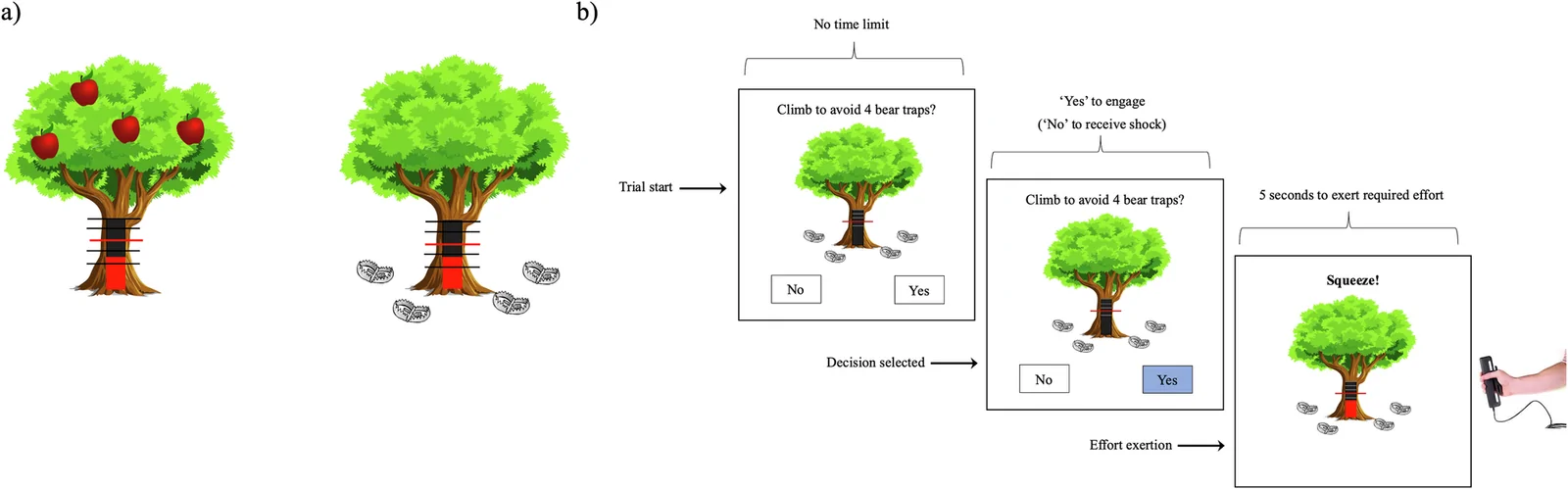
The effort-based decision-making (EBDM) task. **a** On each of the 156 trials, a tree illustrated the level of required effort (ladder rungs) and stake (apples to be retrieved or bear traps to be avoided, respectively). There were five levels of both required effort and stake. For effort, levels were represented by ladder rungs, with the highest level calibrated to 85% of each individual’s maximum force grip (sustainable maximal voluntary contraction; see Methods). Stake level was represented by an increase in the number of apples or bear traps and converted to magnitude of financial gain (for the reward version) or level of electrical stimulation (for the aversive version). See Methods for details. **b** On each trial, participants decided whether or not to “climb” the tree (i.e. to engage with the trial) or to forfeit the potential reward (on the reward version of the EBDM task) or receive the electrical stimulation (on the aversive version of the EBDM task). If participants chose to engage, they were given 5 s to exert the required force via a handgrip dynamometer. To be successful, the required force had to be reached and maintained for a minimum of 500 ms. At the end of each trial the outcome was immediately presented (points added following successful effort exertion on the reward task version; electrical stimulation administered following non-engagement or unsuccessful effort exertion on the aversive task version). See Methods for further details.

On each trial, an image of a tree was displayed (see Fig. 1). The trunk of the tree contained an empty vertical rectangle, intersected by 5 horizontal lines in the upper half of the rectangle representing “ladder rungs” to which the participant might be asked to climb. The top of the box (i.e., the top ladder rung) represented the participant’s sustainable maximum voluntary contraction (SMVC), which was set according to the “calibration of force” section above. Each lower rung then represented a 10% reduction in force, with the rungs visually spaced linearly in the top half of the box to illustrate 60%, 70%, 80%, 90%, and 100% of the SMVC.

On each trial, one of five possible levels of reward was offered by showing 1, 4, 8, 12, or 16 apples displayed in the tree. In addition, a ladder rung was highlighted to indicate the amount of force (60%, 70%, 80%, 90%, or 100% of the SMVC) that would be required to successfully climb the tree and gain the apples for that trial. A participant was given 5 s to decide whether or not to climb the tree to obtain the reward. If they declined a trial, they received 0 apples, and proceeded to the next trial. If they accepted the trial, they were given 5 s to climb to the appropriate ladder rung by applying a sufficient amount of grip force to the dynamometer. Real-time feedback of the grip was shown as a red color filling the rectangle displayed on the tree as the participant squeezed. If the participant held the force at or above the appropriate ladder rung for at least 0.5 s, they obtained the apples at the end of the trial and were shown a message that indicated they had added the specified number of apples to their basket. Otherwise, they gained zero apples, and a message was displayed indicating that they had not succeeded. Participants were informed that they would receive a bonus based on how many apples they collected during the task but were not informed of the conversion rate and were not shown their balance of apples already gathered over time. At the end of the task, the bonus was calculated by prorating the maximum bonus of $7.50 according to the number of apples successfully gathered.

#### The effort-based decision-making task: threat avoidance version

The threat avoidance version of the EBDM task was structurally parallel to the reward pursuit version, but here participants chose whether to climb trees in order to escape varying numbers of bear traps around the base of the tree (1, 4, 8, 12, or 16 bear traps; no apples shown) which represented various levels of electrical stimulation (see Fig. 1). The trial structure, with five levels of effort level and five levels of stimulation was identical to that for reward pursuit. Participants were informed that their subjective calibration of electrical stimulation levels would be translated into the number of bear traps shown, and that higher numbers of bear traps indicated higher levels of stimulation but were not specifically informed of the conversion mapping. This mapping was set such that the 5 distinct numbers of bear traps corresponded to subjectively rated stimulation levels 2, 4, 5, 6, and 7 for the given participant. Trial timings and force requirements were as described for the reward pursuit version of the EBDM task. If participants elected not to exert effort on a given trial or did not manage to maintain the required level of effort for 500 ms they received the level of electrical stimulation associated with that trial.

### Computational modeling of behavior

We modeled participants’ performance on the reward pursuit and threat avoidance versions of the EBDM task using a class of subjective value (SV) models that capture participants’ valuation of both stake (level of reward or threat) and effort. We began with a model (Model #1) which considered only stake level, effort level, and general proclivity to engage, or decline to engage, in effortful pursuit of reward or avoidance of threat, respectively. We then incrementally augmented the model with parameters of known behavioral or theoretical interest. At each stage of augmentation, we assessed model fit using the Watanabe-Akaike Information Criteria (WAIC) [51], a method which assesses comparative model fit while penalizing for model complexity in a hierarchical Bayesian fitting framework. We retained parameters that improved model fit (see Supplemental materials: Computational models tested and Table S2.) To be able to test equivalent hypotheses for reward pursuit and threat avoidance we included separate parameters for reward and threat trials in a single joint model, and either kept or rejected a given parameter for both trial types based on change in model performance.

Since our main hypotheses concerned the effect of individual differences in latent dimensions of negative affect on effortful choice behavior, each model included *β* parameters which assessed the influence of participant factor scores on each group-level model parameter distribution. Following [37], these were formulated as parameterized offsets to each group level parameter mean *μ* based upon individual differences in factor scores. In addition, for each threat parameter, we modeled a group-level offset parameter for baseline level of shock defined for each participant as their “level 1” (denoted L1mV) to control for potential differences in baseline shock sensitivity (see Equation 1b for an example).

### Hierarchical Bayesian parameter estimation

We used a hierarchical Bayesian sampling approach to infer posterior distributions for model parameters at the individual and group level as well as to assess the effects of participant-wise scores on latent factors of negative affect. Models were fit using MCMC sampling using the rstan package [52] in R. For each model, four chains of posterior sampling were run, each with 5500 total iterations and 3500 “burn-in” iterations. Convergence of the sampling was determined by visual inspection of MCMC sampling chains and inspection of the Gelman-Rubin statistics for parameters. Gelman-Rubin statistics provide a measure of the consistency of parameter variance within sampling chains versus between sampling chains. Values very close to 1.0 are generally considered ideal as they indicate high reliability of variance sampling across independent Markov chains. 99.5% of Gelman-Rubin statistics across 936 measures of convergence were between 0.999 and 1.01 in our final model. 0.5% of the statistics were between 1.01 and 1.07, and none was above 1.07. All of the group-level (*β*) parameters quantifying the influence of latent factors of negative affect on task performance which were used for testing our main hypotheses had Gelman-Rubin statistics between 1.000 and 1.006, indicating that all had high convergence reliability. The inclusion of the group-level (*β*) parameters in the hierarchical inference allowed the uncertainty of each result to be directly quantified in the context of the entire joint posterior distribution [53]. Distinct parameter distributions for reward pursuit and threat avoidance were inferred within a single hierarchical Bayesian sampling framework such that the joint posterior distribution accounted for both task variants.

Each participant-level parameter was assumed to be drawn from a Gaussian group-level distribution for that parameter described as ~Normal(μ, σ^2^). Latent dimension factor scores were parameterized as offsets to each group distribution mean to assess the impact of individual differences in these factor scores on model parameters capturing the computations underlying task performance (see Eq. 1a, b below). Hyperparameters were given uninformative priors of μ ~ Normal(0,10) and σ ~ HalfNormal(2). Latent dimension offset parameters were given uninformative priors of *β* ~ Normal(0,10).

As an example, shown below is the fully expanded group-level distribution for the linear stakescale parameter. This example shows the separation of parameters for reward-pursuit and threat-avoidance trials and the individual difference offset parameters (*β*) for each factor score (and for baseline level of electrical stimulation for threat avoidance trial parameters). Analogous expansions for all model parameters were included for all models (“ptp” = participant).

1a $${\gamma }_{\mathrm{\_rew\_ptp}} \sim \mathrm{Normal}({{\rm{\mu }}}_{\mathrm{\gamma \_rew}}+{\beta }_{\gamma \_rew\_G}* {G}_{ptp}+{\beta }_{\gamma \_rew\_F1}* {F}_{1\_ptp}+{\beta }_{\gamma \_rew\_F2}* {F}_{2\_ptp}+\,{\beta }_{{\gamma }_{\_rew\_F{3}}}* {F}_{3\_ptp}+{\beta }_{\gamma \_new\_F4}* {F}_{4\_ptp}+{\beta }_{\gamma \_rew\_F5\_neg}* {F}_{5\_neg\_pt{p}_{,}}{\sigma }_{{{\rm{\gamma }}\_rew}^{2})}$$

1a $${{\rm{\gamma }}}_{{-}^{{\rm{threat}}}{-}^{{\rm{ptp}}}} \sim {\rm{Normal}}({{\rm{\mu }}}_{{\rm{\gamma }}{\rm{\_threat}}}+{{\rm{\beta }}}_{{\rm{\gamma }}{\rm{\_}}{threat}{\rm{\_}}G}* {G}_{{ptp}}+{{\rm{\beta }}}_{{\rm{\gamma }}{\rm{\_}}{threat}{\rm{\_}}F1}* {F}_{1{\rm{\_}}{ptp}}+{{\rm{\beta }}}_{{\rm{\gamma }}{\rm{\_}}{threat}{\rm{\_}}F2}* {F}_{{2}_{{ptp}}}+\,{{\rm{\beta }}}_{{{{\rm{\gamma }}}_{{\rm{\_}}}}_{{{threat}}_{F3}}}* {F}_{{3}_{{ptp}}}+{{\rm{\beta }}}_{{{\rm{\gamma }}{\rm{\_}}}_{{{threa}{t}_{{\rm{\_}}}}_{F4}}}* {F}_{{4}_{{{\rm{\_}}}_{{ptp}}}}+{{\rm{\beta }}}_{{{\rm{\gamma }}}_{{threa}{t}_{F{5}_{{neg}}}}}* {F}_{{5}_{{{\rm{\_}}}_{{ne}{g{\rm{\_}}}_{{ptp}}},}}+{{\rm{\beta }}}_{{y}_{L1{mV}}}* {L1{mV}}_{{ptp}},{\sigma }_{{{\rm{\gamma }}{\rm{\_}}{rew}}^{2})}$$

### Model #1: SV Model

For the simplest model (Model #1), participants’ choices were assumed to be influenced by the level of stake on offer (*γ*) and the level of effort required (κ) for a given trial (_t_). We also included a baseline bias parameter (*b)* which captured individuals’ general propensity to decline to exert effort; this was applied separately from subjective net value. Model #1 therefore included inference over 6 total participant-level parameters: 3 (*γ*_reward_, *κ*_reward_, *b*_reward_) for reward pursuit trials and a parallel 3 for threat avoidance trials (*γ*_threat_, *κ*_threat_, *b*_threat_). A logistic function was used to map each participant’s valuation of the trial to a probability of trial acceptance, and the (yes/no) choice to engage on each trial was assumed to follow a Bernoulli distribution at that probability.

The subjective net value (SNV) for trial *t* for Model #1 was calculated as:

2a $${{SNV}}_{t}={\rm{\gamma }}\left({{Stake}}_{t}\right)-k({{effort}}_{t})$$

The probability that a participant chose to engage on the trial was then given by:

2b $${{\rm{P}}({\rm{Engage}})}_{{\rm{t}}}={(1+\exp (-\left[{{\rm{SNV}}}_{{\rm{t}}}-b\right]))}^{-1}$$

Note that the placement of the parameters was a matter of chosen convention; for example, the baseline offset parameter *b* could be moved from the choice probability formula in 2b to the SNV calculation in 2a without changing the model in any way. We chose to characterize SNV as relating only to stake level and effort level for conceptual clarity.

The group-level mean *μ* and standard deviation *σ* for *γ*_,_ *κ* and *b* were inferred jointly with the participant-level parameters. At the same time, parameter values were also inferred for group-level offsets *β* applied to the group-level distributional means (μ) to capture the influence of participants’ scores on each latent factor from the winning bifactor model (G, F1, F2, F3, F4, and F5_reversed). For the threat-avoidance version of the EBDM task, an additional group level offset was applied to each of the group-level distributional means (μ) to account for any influence of inter-subject differences in objective electrical stimulation level used for ‘level 1’ shock (L1mV). Model #1 therefore included inference over 51 group-level hyperparameters as follows: group-level *μ* and σ for each of the 6 participant-level parameters (12); group-level offsets *β* for each BF5 factor score (G, F1, F2, F3, F4, and F5_reversed) applied to each group-level distributional mean *μ* (36); and group level offsets *β* for baseline electrical stimulation level (L1mV) applied to each threat avoidance parameter group-level distributional mean *μ* (3).

Model #1 was used as a basis for comparison of model fit for subsequent models, with lower WAIC indicating better fit to the data. Following Model #1, we considered additional variables that might affect the subjective value computation, increasing the complexity by including one additional participant-level parameter for reward pursuit trials and the equivalent parameter for threat avoidance trials at each stage (see Supplemental materials: Computational models tested).

#### Model validation: simulated data

To validate the winning model, we simulated four data sets of 50 participants each based on the generative winning model using participant posterior mean parameter values and compared actual participant behavior to the final simulated data. This provides a test of whether the winning model can produce the pattern of choices by actual participants. See Supplemental Materials: model validation and Figure S2.

#### Model validation: parameter recovery

We tested our ability to accurately recover parameter values by fitting the winning model (Model #6) to the simulated participant data sets described above. We then compared the true (known) parameter values to the model-fit (recovered) parameter values. In hierarchical Bayesian inference, a general goal for parameter recovery is that 95% of the recovered posterior parameter distributions include the true parameter value in their 95% highest density posterior interval. See Supplementary Materials: model validation and Figure S3 for parameter recovery results.

#### Model validation: model recovery

As a test of model recovery, we selected four models (the winning model plus three of the non-winning models) from among those that best fit task performance and simulated four separate data sets of 50 participants each for each of these models (using the participant posterior parameter means from each respective model). Each of these 16 simulated data sets was then hierarchically fit by all four models, and we assessed which model fit each data set best based on the lowest WAIC measure across models for that data. This tests the ability of the fitting procedure to correctly identify the model which generated the data as the “best fit” for that data. See Supplemental Materials: model validation and Figure S4 for model recovery results.

#### Model explanatory power

To assess the explanatory power of the winning model of choice behavior, we performed a Bayesian R-squared analysis [54] using the R stan [52] and rstantools [55] packages. Here the generated quantities block of the model was modified such that the sampling estimate of choice for each trial for each participant was generated for every posterior sample draw. Posterior post-burn-in samples for choice estimates were then extracted from the stan fit object, and the Bayes_R2 function was applied to estimate the model’s Bayesian R-squared value across posterior draws (the median value across posterior sample draws is used to obtain the Bayesian R-squared estimate for the model). This provides an estimate of total behavioral variance in task performance explained by the winning model.

To assess the amount of variance in trait affect, as captured by the general and specific latent factors, that was explained by the parameters of the winning model, we performed linear regression for each latent factor score using reward pursuit and threat avoidance model parameters from the winning model (Model #6) as predictors. Baseline parameter values prior to adjustment for parameter effects of latent trait scores were used, and the resulting R-squared results are shown in Figure S5a. To assess predictive significance, n = 1,000 simulations were run predicting randomly permuted scores. Significance of actual predictive values were calculated using the percentage of permutation samples exceeding the actual predictive value, shown in Figure S5b.

## Results

### Bifactor modeling of latent sub-dimensions of negative affect

Using the large online datasets, we performed exploratory and confirmatory bifactor analyses specifying one general factor and varying the numbers of specific factors from two through seven.

**Fig. 2:**
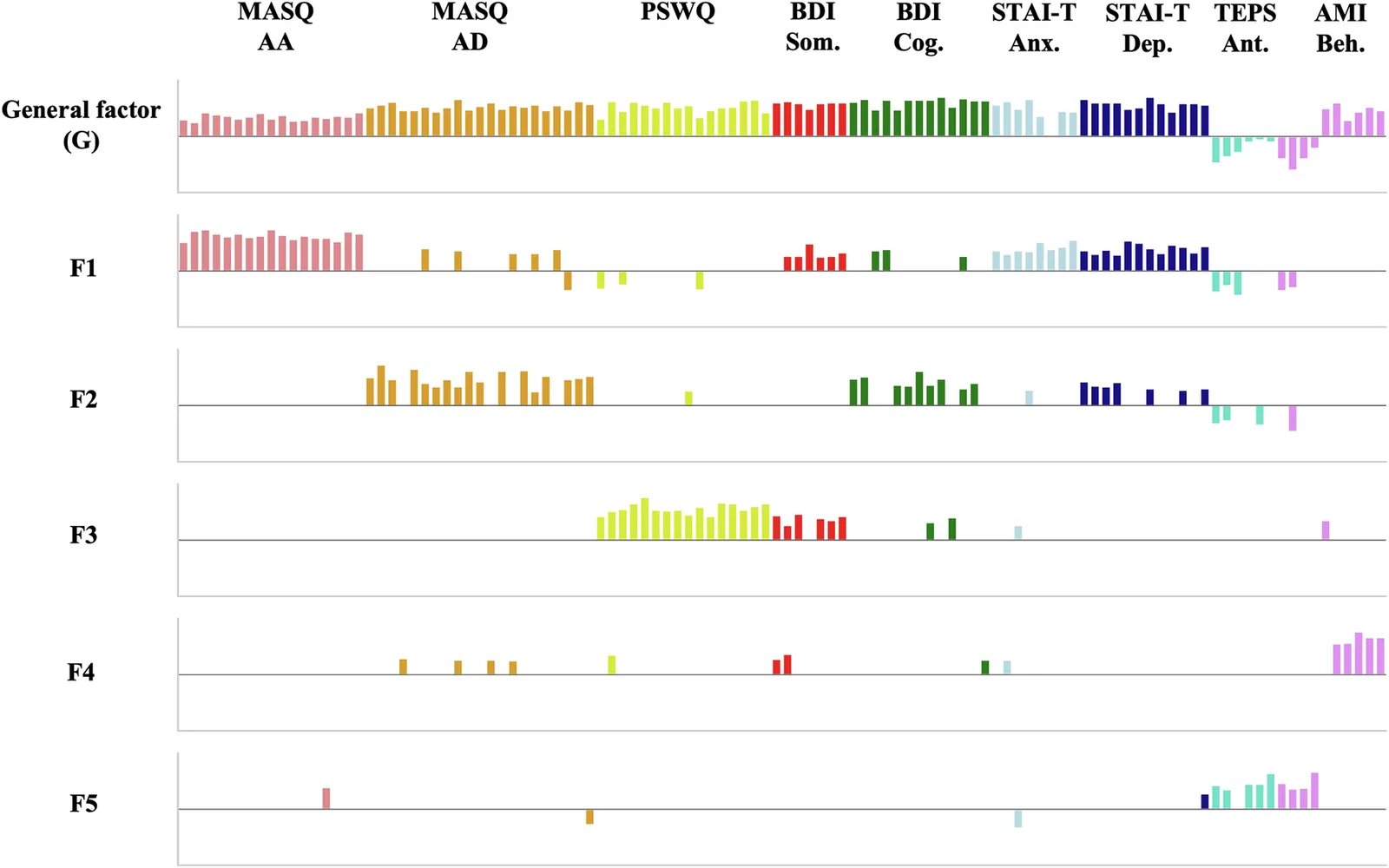
Bifactor analysis of item-level questionnaire data. Item loadings for the winning bifactor model (BF5) with one general and five specific factors. Questionnaires from left to right: Mood and Anxiety Symptom Questionnaire (MASQ) [25] anxious arousal (AA) and anhedonic depression (AD) subscales; Penn State Worry Questionnaire (PSWQ) [39]; the Beck Depression Inventory [38] somatic (Som.) and cognitive (Cog.) subscales; the State Trait Anxiety Inventory - Trait version (STAI-T) [40] anxiety (Anx.) and depression (Dep.) subscales [41]; Temporal Experience of Pleasure [42] anticipatory anhedonia subscale (TEPS Ant.); Apathy Motivation Index [23] behavioral subscale (AMI Beh.).

The bifactor model with five specific factors (BF5) provided the best combination of exploratory model fit (BIC = 4468.38) while maintaining a confirmatory fit index (CFI) above 0.95 in the held-out data (CFI = 0.953, RMSE = 0.101); see Fig. 2, Figure S6 and Table S2. Specifically, a comparison of the Bayesian Information Criteria (BIC), which assesses model fit while penalizing for complexity, showed continued improvement in exploratory model fit as additional specific factors (up to seven, the maximum tested) were included. However, when more than five specific factors were included, the confirmatory fit metrics fell below 0.95, indicating that increasing the number of specific factors above five resulted in a worse fit in the confirmatory dataset, and suggesting that more complicated models were overfit to the exploratory data. The winning model also provided greater factor interpretability than models with fewer specific factors.

**Fig. 3:**
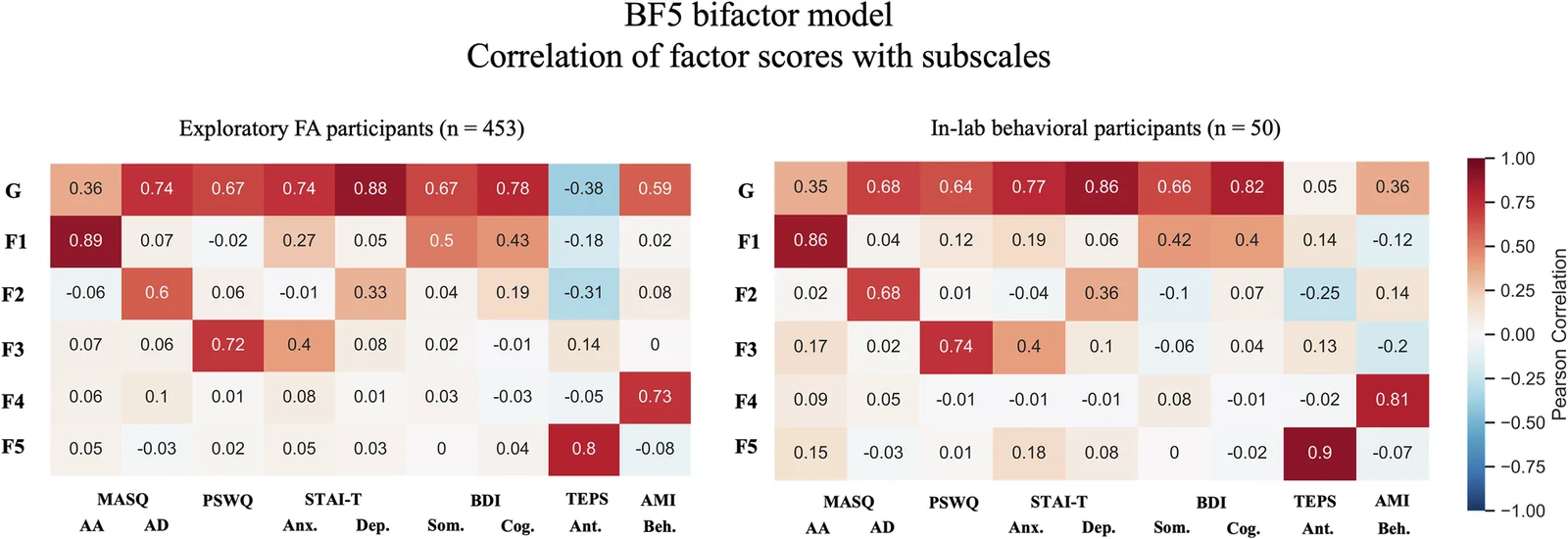
Correlations between factor scores from the winning bifactor model (BF5) and questionnaire scale summary scores. The left-hand matrix shows correlations between factor scores and questionnaire summary scores for online participants whose data were used in the exploratory factor analyses (n = 453); the right-hand matrix shows correlations for in-lab participants who completed the effort-based decision-making tasks (n = 50). Summary scores (x axis) were calculated for the same questionnaire subscales as reported in Fig. 2. The y axis specifies each of the factors from the winning BF5 bifactor model (G is the general factor, F1 to F5 are the five specific factors). MASQ AA: MASQ anxious arousal subscale; MASQ AD: MASQ anhedonic depression subscale; STAI-T Anx.: STAI-T anxiety subscale; STAI-T Dep.: STAI-T depression subscale); BDI Som: BDI somatic subscale; BDI Cog: BDI cognitive subscale; TEPS Ant.: TEPS anticipatory hedonia subscale; AMI Beh.: AMI behavioral subscale.

**Fig. 4:**
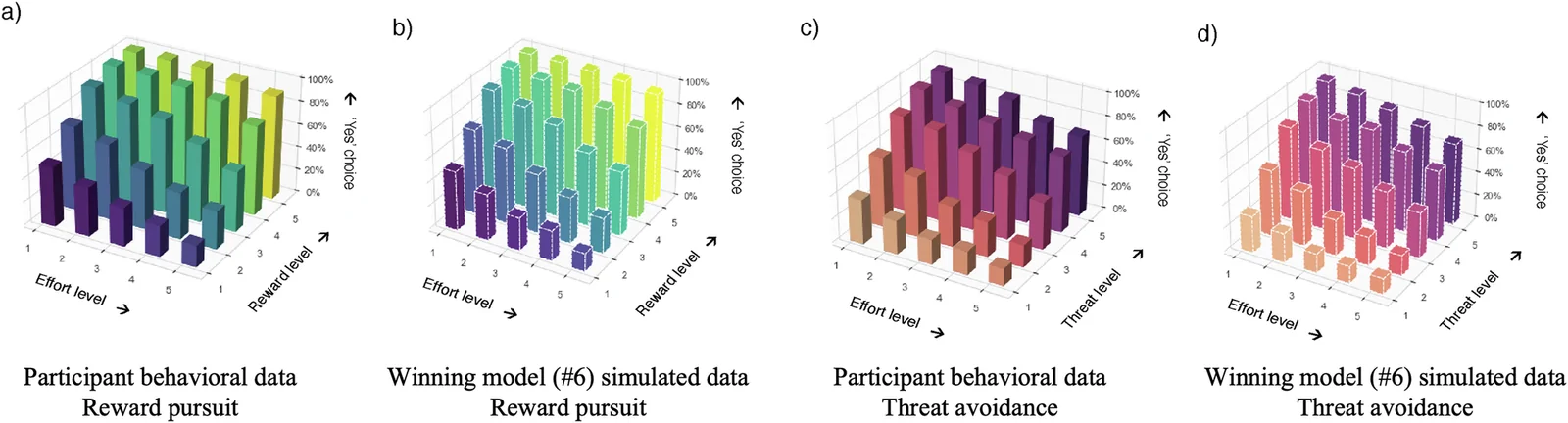
Behavioral choices on the reward and aversive versions of the EBDM task as made by participants and as simulated using the winning computational model. **a**) This 3 d plot shows participants’ mean group-level behavior (probability of trial engagement, i.e. ‘yes’ choice) for the reward version of the effort-based decision-making (EBDM) task as a function of effort level and stake (financial reward) level. **b**) This 3 d plot shows simulated choice behavior (probability of trial engagement) on the reward version of the EBDM task as generated by the winning computational model (Model #6). **c**) This 3 d plot shows participants’ mean group-level behavior (probability of trial engagement) on the aversive version of the EBDM task as a function of effort level and stake (electrical stimulation) level. **d**) This 3 d plot shows simulated choice behavior (probability of trial engagement) on the aversive version of the EBDM task as generated by the winning computational model (Model #6). See Figure S2 for additional 2 d plots of simulated choice behavior.

The item loadings of the winning bifactor model (BF5) were used to calculate factor scores for each participant in the exploratory data set (see Methods). We examined the correlation of participants’ scores for each factor from the winning model with their summary scores for the standardized questionnaire scales (or subscales) to provide additional insight into factor interpretation (Fig. 3, left panel).As anticipated, we observed strong correlations between scores on the general factor and summary scores for most of the scales (noting that the TEPS anticipatory hedonia scale is coded oppositely from the other scales, such that a higher TEPS score indicates greater positive affect). In addition, scores on each specific factor were strongly correlated with summary scores for one or more questionnaire scales. We performed the same correlational analysis between scale summary scores and BF 5 model factor scores for the in-lab behavioral participants who completed the EBDM tasks to ensure consistency in factor interpretation. The correlations for the in-lab behavioral group (Fig. 3, right panel) showed notable similarities to those observed for the EFA group. Based upon the item loadings and correlations with questionnaire scores, we characterized the five specific factors from the BF5 model as follows: Factor 1: Mixed anxiety and depression; Factor 2: Anhedonic depression; Factor 3: Worry; Factor 4: Apathy; Factor 5: Anticipatory hedonia. These labels are subjective and are intended to reflect the predominant symptom variance captured by each specific factor (Fig. 4). Unlike the other scales, higher scores on TEPS indicate greater positive affect. For consistency, we reversed participant scores on F5 (F5-reversed) for all subsequent analyses such that higher scores on F5-reversed indexed greater anticipatory anhedonia.

### Effort -based decision-making (EBDM) task performance

On every trial of the EBDM task, participants had to decide whether to exert effort or not. In the reward version of the EBDM task, a successful outcome entailed obtaining the tree’s apples (Fig. 1a). In our new, structurally equivalent, aversive version of the task, participants avoided “bear traps” by climbing the tree (Fig. 1b). Here, a successful outcome entailed avoiding electrical stimulation. The number of apples or bear traps, respectively, indicated the relative magnitude of monetary gain or electrical stimulation for the trial, while the red ladder rung on each trial (Fig. 1a) indicated the effort required to succeed. There were five levels of stake and five levels of effort in each task version with six trials at every stake by effort combination (plus six additional trials, see Methods). Objective effort requirements and electrical stimulation intensities were calibrated for each participant. Before fitting computational models to participants’ performance of the reward and aversive versions of the EBDM task, we visualized the effect of our experimental manipulations on participants’ trial-wise decisions to exert or withhold effort to obtain reward or to avoid threat (electrical stimulation). Participant behavior showed sensitivity to increases in reward level, threat level, and effort level, as illustrated in Fig. 4a and c.

### Logistic regression analyses of participant choice behavior

We next performed a simple logistic regression analysis of participants’ choices to engage in effortful trials. For both the reward and aversive EBDM task versions, choices were significantly modulated by trial-wise stake and effort level, with increasing stake levels increasing the probability of participants choosing to attempt to “climb” the tree (i.e. to engage with the trial); reward pursuit: stake level coefficient = 1.13, 95% confidence interval (CI)= [1.075,1.186], Fig. 4a, Figure S2a; threat avoidance: stake level coefficient =0.774, 95% CI = [0.733,0.815], Fig. 4c, Figure S2c. Meanwhile, increasing effort levels decreased the probability of participants choosing to “climb” (trial engagement); reward pursuit: effort level coefficient = -0.526, 95% CI = [-0.571,-0.480], Fig. 4a, Figure S2e; threat avoidance: effort level coefficient = -0.430, 95% CI = [-0.468,-0.391], Fig. 4c, Figure S2g. These choice patterns are consistent with those reported in prior studies of EBDM using the original reward task version [21].

### Computational modeling of EBDM task performance

We used subjective value-based computational modeling to explore the influence of specific predictors on participants’ choices. We considered six alternative models of choice, fitting each model in a hierarchical Bayesian framework (see Methods), and assessed the fit of each model to the data using the Watanabe-Akaike Information Criteria (WAIC, also known as the Widely Applicable Information Criteria) [51]. The different models attempted to capture participants’ likelihood of engaging in a trial using distinct parametrizations of the underlying computations (see Supplemental Materials for full details of the six models considered and Table S2 for model comparison). The model which provided the best fit to the data (Model #6; WAIC = 5709.68, se = 119.71) included parameters for linear (*γ*) and nonlinear (*θ*) scaling of stake level; linear scaling (*κ*) of required effort level; baseline bias against effort exertion (*b*); a stake per effort interaction parameter (*δ*); the effect of decreasing engagement over the course of the task (*τ*); a parameter to account for probability of success at a given level of effort (ρ); and a parameter to characterize a participant’s tendency to perseverate (i.e., to repeat the previous response regardless of changes in stake or effort level, sometimes referred to as ‘sticky choice’) (η). Separate parameter distributions were inferred for behavioral choices on the reward and aversive versions of the EBDM task. Parameters for both reward pursuit and threat avoidance were fit within a single hierarchical Bayesian sampling framework such that the joint posterior distribution accounted for both task versions. The winning model (Model #6) explained an estimated 69% of behavioral variance in task performance (Bayesian R-squared = 0.69).

For the winning model (Model #6), the subjective net value (SNV) of the offer on trial *t* was calculated as a function of the tradeoff between stake (i.e. reward or threat level) and effort as follows:

3a $${{\rm{SNV}}}_{t}={{{\gamma}}({{Stake}}_{t})}^{\theta}{\;{\mbox{-}}}\;k\left({{Effort}}_{t}\right)+{{\delta}}({{Stake}}_{t}/{{Effort}}_{t})$$

The probability that a participant would engage on a given trial *t* was calculated as a logistic function of the SNV while also allowing other factors (see above and Methods) to bias choice to engage as follows:

3b $${{\rm{P}}({\rm{Engage}})}_{{\rm{t}}}=(1+\exp \left(-\left[{{\rm{SNV}}}_{{\rm{t}}}-{\rm{\tau }}\left({{\rm{Trial}}}_{{\rm{t}}}\right)+{\rm{\rho }}\left({{\rm{Psuccess}}}_{{\rm{t}},{\rm{e}}}\right)+{\rm{\eta }}\left({{\rm{Choice}}}_{{\rm{t}}-1}\right)-{\rm{b}}\right]\right))$$

To examine how participants’ scores on latent dimensions of anxious and depressive symptoms impacted their choice behavior, we included an “offset” group-level parameter *β* for each factor from the winning bifactor model (BF5) on each choice parameter [37]. This allowed us to estimate directly the strength of the trait effects on choice by jointly inferring posterior distributions for each *β* along with the choice model parameters. A 95% highest density interval (HDI) of the posterior distribution for a given *β* parameter which excluded 0 indicated a significant influence of the factor scores associated with that *β* on the choice parameter.

To validate the winning model, we simulated four data sets of 50 participants each based on the generative winning model using participant posterior mean parameter values and compared actual participant behavior to the final simulated data. The model-simulated data reproduced participant choice behavior (Fig. 4, Figure S2). We performed additional analyses to establish that parameters from the final model could be accurately recovered (see Methods, Supplemental Materials: model validation and Figures S3, S4 for details) and to ensure that there was not high collinearity of participant-level posterior parameter means; see Methods, Supplemental Materials: model validation and Figure S7.

### Group-level results

Before examining the effect of latent factors tapping anxiety and depression on task performance, we assessed group-level model-based results across the reward and aversive versions of the EBDM task. We also examined the relationship between individual differences in EBDM performance across the two versions of the task. Here we present results for select model parameters; for full analyses of all parameters and additional comparison details, see the Supplemental Analyses and Figure S8.

**Fig. 5:**
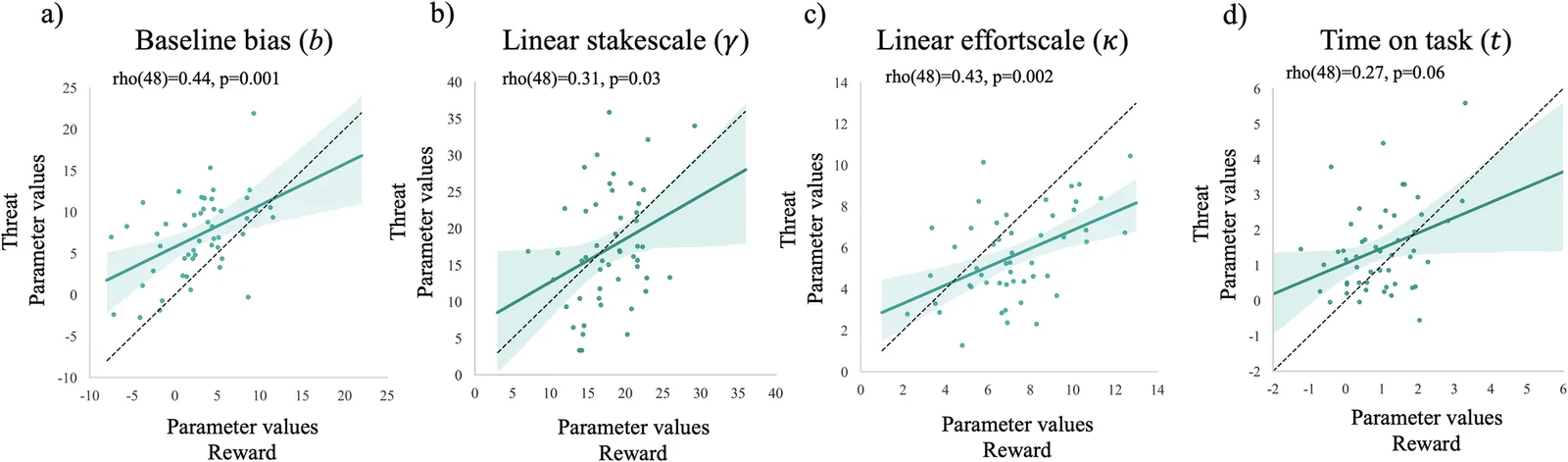
Participant-level correlations between reward and aversive EBDM task version parameter values. Each panel presents participants’ posterior means (n = 50) for a specific parameter from the winning model (Model #6) as fitted to data from the reward pursuit (x axis) and threat avoidance (y axis) versions of the EBDM task. From left to right, parameter posterior means are given for (**a**) baseline bias (higher values indicate reduced willingness to engage in trials and exert effort), (**b**) linear stakescale (higher values indicate greater subjective weight is given to reward or threat level), (**c**) linear effortscale (higher values indicate greater subjective weight is placed on the level of required effort), and (**d**) time-on-task (higher values indicate a larger reduction in trial engagement/willingness to exert effort over the course of the task). The solid line and shaded region show the regression of threat-avoidance parameter posterior means on reward-pursuit posterior means and associated 95% confidence interval. Dashed line shows unity. Spearman correlations (rho) and associated p-values are given above each plot. See Figure S9 for plots for all model parameters.

Group mean values for the baseline bias parameter, which describes baseline bias against trial engagement, revealed a significantly higher bias against engagement for effortful threat avoidance than for effortful reward pursuit (95% HDI of reward mean – threat mean = [-7.61, -1.42]; see Supplemental Analyses for details). Individual posterior mean values for the baseline bias parameter were correlated across reward and aversive task versions (rho(48) = 0.441, p = 0.001), indicating that heightened willingness to engage in reward pursuit was also linked to heightened willingness to engage in threat avoidance across individuals (Fig. 5a; see Supplemental Analyses and Figure S8 for further details).

At the group level, there was no significant difference between reward pursuit and threat avoidance for linear scaling of stake (i.e. sensitivity to stake-level; 95% HDI of reward mean – threat mean =[-2.92,4.64])), while group-level linear scaling of effort (i.e. sensitivity to required effort level) was significantly greater for reward pursuit than threat avoidance (95% HDI of reward mean – threat mean =[4.51,6.84]); see Supplemental Analyses for details. Individual posterior mean values were significantly correlated between reward pursuit and threat avoidance for both linear stakescale (rho(48) = 0.308, p = 0.030; Fig. 5b) and linear effortscale (rho(48) = 0.425, p = 0.002; Fig. 5c), confirming a degree of shared variance across task versions in the extent to which participants’ choices were influenced by stake and effort level. Finally, at a group level, there was no significant difference between reward pursuit and threat avoidance for the time-on-task parameter which captured declining participant willingness to engage in effort over the course of the task (95% HDI = [-1.05,0.02]). In addition, individual posterior mean values parameter values for time-on-task were not significantly correlated across task versions (rho(48) = 0.268, p = 0.06; Fig. 5d).

### Task performance modulation by individual differences in latent negative affect

In the hierarchical modeling framework used, group-level *β* parameters captured the influence of participants’ scores for each factor from the bifactor model of anxious and depressive affect on each parameter of the winning model as fit to participants’ performance on the reward and aversive versions of the EBDM task. By examining the posterior distributions of these group-level weights, we determined the significance of the effects of each of these group-level parameters. See Figure S9 for complete results across all latent factors and model parameters.

### Influences of separate dimensions of depressive affect on effortful reward pursuit

**Fig. 6:**
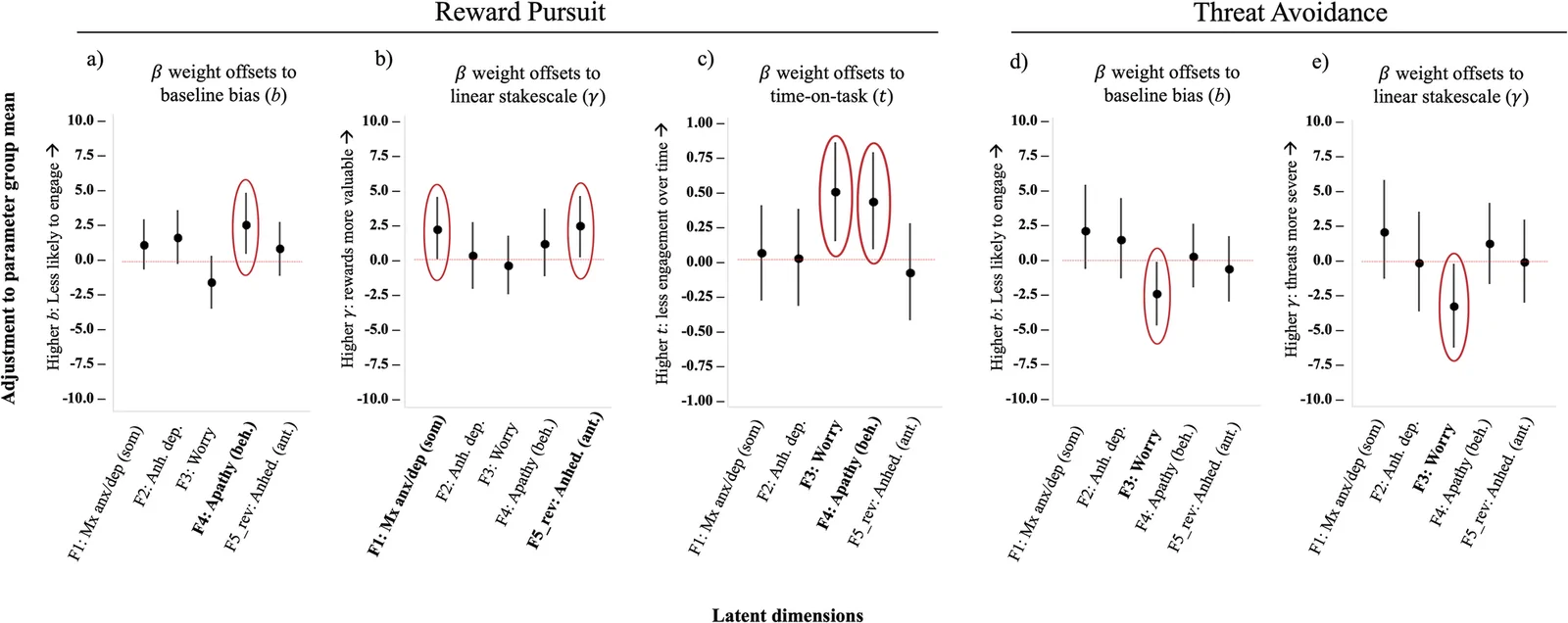
Effect of specific latent factor scores on posterior parameter values for the reward and aversive versions of the EBDM task. Panels show posterior means and 95% highest density intervals (HDI) for the effect of each of the five specific factors from the bifactor analysis of anxious and depressed affect, upon parameter posterior values obtained by fitting the winning model (Model #6) to performance on the reward version of the EBDM task (panels a-c) and the threat version of the EBDM task (panels d-e). Reward EBDM findings. **a** Specific latent factor F4 (behavioral apathy) was the only factor to modulate parameter values for baseline bias with higher F4 scores being linked to a greater general tendency to decline to exert effort across all effort and stake (reward) levels. **b** Modulation of group-level posterior parameter values for the linear effect of stake (γ). Scores on specific factor F5 (reverse scored; anticipatory anhedonia) were associated with greater sensitivity to stake level. A similar effect was also observed for specific latent factor F1 (mixed anxiety and depression). **c** Scores on both specific factor F4 (behavioral apathy) and F3 (worry) were associated with reduced willingness to exert effort as the task progressed. Note, none of the latent specific factors significantly modulated the effect of level of required effort; this was however modulated by scores on the general factor (Figure S9). Aversive EBDM findings. **d** Specific latent factor F3 (worry) was the only factor to modulate parameter values for baseline bias (*b*), with higher F3 scores being linked to a reduced general tendency to decline to exert effort across all effort and stake (threat) levels. **e** Modulation of group-level posterior parameter values for the linear effect of stake (γ). Scores on specific factor F3 were also associated with reduced sensitivity to stake (threat) level when deciding whether to exert effort. There was no significant modulation by latent dimensions of negative affect of any other parameter values (Figure S9).

We first examined the influence of participant factor scores on group-level parameter estimates for data from the reward version of the EBDM task (Fig. 6, Figure S9). Elevated scores on factors capturing heightened behavioral apathy (F4) and anticipatory anhedonia (F5_reversed) were significantly associated with reduced effortful reward pursuit, in line with previous findings. However, each operated on distinct mechanistic components of decision-making.

Specifically, we found that higher scores on the factor capturing behavioral apathy (F4) were associated with a significantly greater bias against engaging in effortful reward pursuit (β_F4___baseline___reward_: *μ* = 2.48, 95% posterior HDI = [0.572, 4.681]; Fig. 6a). Higher scores on F4 were also associated with a significantly larger drop in effortful engagement over time on task as indexed by trial number (β__F4___timeontask_reward_: *μ* = 0.398; 95% posterior HDI = [0.073, 0.735]; Fig. 6c). There was not a significant effect of participant scores on this apathy factor (F4) on parameter estimates capturing sensitivity to the level of stake (β_F4___stakescale___reward_ : *μ* = 1.06, 95% posterior HDI = [-1.158, 3.457]; Fig. 6b) or the level of effort required (β_F4___effortscale___reward_ : *μ* = -0.47, 95% posterior HDI = [-1.636, 0.666]; Figure S9).

In contrast to the findings for the factor capturing behavioral apathy (F4), elevated scores on the specific factor capturing anticipatory anhedonia (F5_reversed) were significantly associated with elevated sensitivity to stake (the level of reward on offer; β__F5neg_stakescale_reward_: *μ* = 2.28, 95% posterior HDI = [0.158, 4.293]; Fig. 6b). This was associated with a pattern in which individuals with higher scores on F5_reversed (i.e., higher anticipatory anhedonia as captured by this specific factor) were less willing to engage in effortful pursuit than those with low anhedonia for low stakes, but equivalently so when the reward was greater (see Figure S10a for illustration). Meanwhile, there was no significant relationship between scores on the specific factor capturing anticipatory anhedonia (F5_reversed) and either baseline bias against effortful reward pursuit (β_F5rev___baseline___reward_: *μ* = 0.87, 95% posterior HDI = [-0.956, 2.757]; Fig. 6a) or change in effortful engagement across trials (β_F5rev___timeontask___reward_: μ = -0.09, 95% posterior HDI = [-0.416, 0.248]; Fig. 6c). Jointly, these results are consistent with distinct mechanisms of operation for different latent dimensions of depressive symptoms upon effortful reward pursuit.

### Influences of anxious affect on effortful reward pursuit

Higher scores on the specific factor tapping worry (F3) were associated with a significantly larger drop in effortful engagement over time (β__F3___timeontask___reward_: μ = 0.464, 95% posterior HDI = [0.123, 0.801]; Fig. 6c). This acts to offset the non-significant baseline tendency of individuals with higher scores on F3 to choose to exert effort across all effort and stake levels (Fig. 6a). This contrasts with the effects of the factor tapping behavioral apathy (F4), where a baseline bias to decline to exert effort is compounded by an effect in the same direction of time on task.

Finally, we also found that higher scores on F1, which captured mixed symptoms of anxiety and depression, were significantly associated with heightened sensitivity to stake (reward) level (β__F1___stakescale___reward_: μ = 2.06, 95% posterior HDI = [0.038, 4.267]; Fig. 6b).

### Effortful avoidance of threat: heightened bias but reduced sensitivity in worriers

We next examined the influence of participant factor scores on group-level parameter estimates for data from the aversive version of the EBDM task (Fig. 6d,e). In line with the hypothesis that anxiety-related components of negative affect might be associated with increased willingness to exert effort to avoid threat, elevated scores on the latent specific factor associated with worry (F3) were significantly associated with a decreased baseline bias against engaging in effortful avoidance of shock receipt (β__F3___baseline___threat_: μ = -2.304, 95% posterior HDI = [-4.585, -0.198]), Fig. 6d. Elevated scores on factor F3 were also significantly associated with reduced sensitivity to stake (β__F3___stakescale___threat_: μ = -3.112, 95% posterior HDI = [-5.948, -0.284]), Fig. 6e, indicating a blunted relationship between increasing threat levels and willingness to exert effort in individuals with high F3 scores. Thus, in the mirror image of the effect of anhedonia in the case of reward, individuals with high worry scores were more willing to engage in effortful avoidance than those with lower worry for low stakes, but equivalently so when the threatened punishment level was high (see Figure S10b).

No other latent dimension of negative affect significantly modulated any of the computations involved in effortful threat avoidance as assessed by our winning model (Fig. 6d,e, Figure S9).

### Relationship between the common factor ‘G’ and effortful decision-making

The higher order factor ‘G’ was significantly associated with a flattened response to increases in required effort level in reward pursuit (β__G___effortscale___reward_: μ = -1.256, 95% posterior HDI = [-2.468, -0.087]). There was no significant association of ‘G’ with any other model parameter in either reward pursuit or threat avoidance; see Figure S9.

## Discussion

To date, human studies examining the relationship between internalizing psychopathology and effort-based decision-making have largely focused upon depression, associated dimensional measures of apathy and anhedonia, and the pursuit of reward. It is difficult to be certain as to the specificity of the reported associations given the lack of work on effortful avoidance of threat, the high comorbidity between anxiety and depression and the substantial shared variance between participants’ scores on scales tapping anhedonia and apathy. We sought to address this in the work reported here.

To address specificity with regards to domain (reward or threat), we created parallel versions of an EBDM task and fit a common computational model to participants’ performance on both task versions to capture the impact of underlying computational processes on choice. To address specificity with regards to different sub-dimensions of internalizing symptoms, we fit a bifactor model to item-level responses from questionnaire measures of anxiety and depression. This enabled us to separate common variance linked to a higher-level ‘general’ factor from that uniquely captured by lower-level specific factors, including, crucially, ones capturing apathy, anhedonia, and worry with shared symptom variance removed. Finally, we brought this together by performing hierarchical Bayesian inference jointly over decision-making parameters and the parameterized effects of individual differences in factor scores.

Our first aim was to determine whether different subdimensions of depressive affect are associated with alterations to the same or different computational mechanisms in the context of effortful pursuit of reward. We found that elevated scores on latent factor F4, associated with behavioral apathy, were linked to an increased baseline bias towards declining effortful reward pursuit as well as a greater diminishment of effortful reward pursuit over the course of the task. In other words, individuals high in behavioral apathy were more generally biased against exerting effort to obtain reward, and this increased as they proceeded further into the task. In contrast, elevated scores on latent factor F5_reversed, associated with anticipatory anhedonia, were linked to heightened sensitivity to increases in stake level, which reflected a reduced willingness to engage in effortful trials for rewards of low magnitude that was overcome at higher reward levels (Fig. 6d). These findings support the contention that distinct latent dimensions of depressive affect might influence effortful reward pursuit through different computational mechanisms. Given that anhedonia and apathy occur together in many, though not all, individuals with depressive disorders, this could potentially lead to a compound effect in such individuals of both a reluctance to engage in effortful reward pursuit in general that increases with the time spent on the pursuit in hand and reduced willingness to pursue rewarding activities unless the reward magnitude is perceived to be of especially high subjective value.

Identifying the relationship between different subdimensions of depressive affect and the specific computational processes contributing to reduced engagement in effortful pursuit of rewarding activities might help in the development of more targeted interventions for individuals experiencing depressive symptomatology. For example, the current findings may suggest that individuals with high anticipatory anhedonia might benefit most from encouragement to revalue activities seen as of low reward value or to test how they feel after engaging in such activities, whereas individuals with high behavioral apathy might benefit from being encouraged to reevaluate or problem solve around the effort required to engage in a rewarding activity. This might extend to predicting which individuals are most likely to benefit from behavioral activation therapy though this is clearly a question for future research. Similarly, in future, it would also be of value to establish the extent to which specific pharmacological interventions increase baseline willingness to engage in effortful pursuit of reward, decrease the drop off in willingness to engage across time or alter the difference in weight given to activities seen as mildly, moderately or extremely pleasurable. Taken together, findings from such studies could guide treatment selection for individuals with different profiles of depressive symptoms.

Our second main aim was to determine whether anxiety-specific affect is associated with differences in effortful decision-making, and if so whether this varies between effortful reward pursuit and effortful threat avoidance. In the effortful threat avoidance task, the latent factor with high loadings on worry-related questionnaire items (F3), was the only latent factor to modulate parameter values significantly. Scores on this factor were associated both with an increased baseline willingness to exert effort to avoid threat, and decreased sensitivity to increases in threat level, suggesting that individuals with high levels of anxiety-specific affect show willingness to exert effort to avoid even low levels of threat. Here, our work extends and complements findings from studies using instrumental threat avoidance paradigms [56, 57] by novelly examining the extent to which anxious individuals’ avoidance of threat is modulated by the level of effort required to engage in avoidance or by the severity of the threat to be avoided.

The influence of F3 scores on parameter values for the reward variant of the EBDM task showed a notably different pattern. There was no significant modulation by F3 scores of either baseline bias to engage in effortful pursuit of reward or sensitivity to increases in reward level (Figure S9) and elevated F3 scores were actually significantly associated with greater diminishment of effortful reward pursuit over the course of the task (Fig. 6). This reveals a distinct and opposing relationship between high anxious affect and propensity to exert effort in avoidance of threat versus pursuit of reward. The reward and threat versions of the EBDM tasks were completed on different days so there is unlikely to be a direct trade-off in effort exertion between the two tasks; instead, this could potentially reflect a more trait-like preference as to under which circumstances to exert effort.

Our third aim was to determine whether anxious versus depressive affect differentially modulate effortful decision-making in reward pursuit and threat avoidance. When we consider the full picture across both effortful pursuit of reward and avoidance of threat, we see clear differential influences of depression and anxiety-specific affect. High scores on latent dimensions tapping symptoms of apathy and anticipatory anhedonia were associated with different facets of reduced willingness to exert effort in pursuit of reward. No significant relationships were observed between scores on these dimensions and any of the computations underlying exertion of effort to avoid threat. In contrast, elevated scores on the latent dimension tapping worry were linked with increased effortful avoidance of threat and reduced effort in pursuit of reward as a function of time on task.

In addition to assessing the impact of latent factor scores on the behavioral model parameters, we can also ask how much variance in trait affect, as captured by the general and specific latent factors, can be explained by the parameters of our winning model. The results are presented in Figure S5a, with results of permutation-based significance testing shown in Figure S5b. For each latent factor, at least 64% of the variance in factor scores, across individuals, was explained by model parameter values, with this rising to 90% for apathy. In all cases this was significantly greater than chance, all ps < .001.

A limitation of our current study is that our sample size was constrained by conducting the study in person as opposed to online to be able to examine EBDM in avoidance of threat as well as in pursuit of reward. In the hierarchical modeling framework used, the inference over the joint posterior automatically captures increased uncertainty due to the inclusion of multiple parameters and regressors of interest making it less likely to find statistically credible posterior 95% HDIs and reducing the likelihood of type I error [53]. Nonetheless, it would be of value to replicate the current findings in a larger sample, with pre-registration of analyses and predicted results. In addition, our study sample comprised young adults pre-screened to span a wide range of sub-clinical symptoms of anxiety and depression. Examination of the mean scores on the questionnaire measures administered in the same sessions as task performance (Table S1) reveals, as expected, that these are lower than would be obtained in a clinical sample diagnosed with anxiety or depressive disorders. It will therefore be important for future work to assess whether the findings reported here also hold in clinical groups seeking treatment for anxiety and/or depression.

We predicted distinct and potentially opposing correlates of anxious and depressive affect with performance on our two EBDM task versions, and did not have strong a priori predictions as to the correlates of the general factor ‘G’ (which we have investigated previously [37]) or indeed the specific factor F1, which showed high loadings for items from the MASQ anxious arousal subscale along with items belonging to both somatic and cognitive subscales of the Beck Depression Inventory. Scores on F1 were linked to increased sensitivity to stake level in reward pursuit, potentially reflecting reduced willingness to exert effort in pursuit of smaller sized rewards, in a similar pattern to that observed for the latent factor capturing anticipatory anhedonia (Fig. 6). Meanwhile, scores on the general factor were linked to a flattened response to increases in required effort level in reward pursuit (Figure S9). For one or both of these factors, there might be a complex interplay of low mood and somatic symptoms of depression being offset by elevated anxious affect. It would be important to replicate these results prior to interpreting them more extensively. However, we note that the co-presence of anxious and depressed affect can be a proxy for symptom severity. Future work indexing not only symptom breadth but also level of functional impairment will be of value in enabling this to be addressed more directly.

In summary, our findings suggest that different subdimensions of depressed and anxious symptoms act via computationally distinct pathways in their influences on effortful decision-making in humans. This points to the value of computationally modeling performance on tasks tapping effortful avoidance of threat as well as effortful pursuit of reward to understanding the distinct computational processes that potentially give rise to common behavioral phenotypes. Currently, the majority of individuals presenting with anxiety and depressive disorders are treated through a trial-and-error approach with the same default first-line cognitive and pharmacological treatments of choice, despite potentially very different symptom profiles. More nuanced information about the relationship of specific symptom profiles with specific deficits in the computations underlying willingness to exert effort to pursue reward or avoid threat may allow for more theoretically driven approaches to be developed.

## Supplementary information

Supplemental Material

## Data Availability

Data and code are available at: https://osf.io/ub8y2.
