# Supplementary material for: Latent subdimensions of anxiety and depression differentially influence exertion of effort in pursuit of reward versus avoidance of threat
Source: Transl Psychiatry. 2026 Jun 30;16:472. doi: 10.1038/s41398-026-04194-0 (PMC13586378; doi:10.1038/s41398-026-04194-0)
Supplement: Supplementary file 1 [file 41398_2026_4194_MOESM1_ESM.pdf]

Supplemental Materials

Computational models tested.

Model #1: SV Model

For the simplest model (Model #1), participants' choices were assumed to be influenced by the level of stake on offer (γ) and the level of effort required (κ) for a given trial ($_t$). We also included a baseline bias parameter (*b)* which captured individuals' general propensity to decline to exert effort; this was applied separately from subjective net value. Model #1 therefore included inference over 6 total participant-level parameters: 3 (γ_{reward}, κ_{reward}, b_{reward}) for reward pursuit trials and a parallel 3 for threat avoidance trials (γ_{threat}, κ_{threat}, b_{threat}). A logistic function was used to map each participant's valuation of the trial to a probability of trial acceptance, and the (yes/no) choice to engage on each trial was assumed to follow a Bernoulli distribution at that probability.

The subjective net value (SNV) for trial *t* for Model #1 was calculated as:

$$SNV_t = \gamma(Stake_t) - \kappa(Effort_t) \quad 2(a)$$

The probability that a participant chose to engage on the trial was then given by:

$$P(Engage)_t = (1 + \exp(-[\ SNV_t - b]))^{-1} \quad 2(b)$$

Note that the placement of the parameters was a matter of chosen convention; for example, the baseline offset parameter *b* could be moved from the choice probability formula in 2b to the SNV calculation in 2a without changing the model in any way. We chose to characterize SNV as relating only to stake level and effort level for conceptual clarity.

The group-level mean μ and standard deviation σ for γ, κ and *b* were inferred jointly with the participant-level parameters. At the same time, parameter values were also inferred for group-level offsets β applied to the group-level distributional means (μ) to capture the influence of participants' scores on each latent factor from the winning bifactor model (G, F1, F2, F3, F4, and F5_reversed). For the threat-avoidance version of the EBDM task, an additional group level offset was applied to each of the group-level distributional means (μ) to account for any influence of inter-subject differences in objective electrical stimulation level used for 'level 1' shock (L1mV). Model #1 therefore included inference over 51 group-level hyperparameters as follows: group-level μ and σ for each of the 6 participant-level parameters (12); group-level offsets β for each BF5 factor score (G, F1, F2, F3, F4, and F5_reversed) applied to each group-level distributional mean μ (36); and group level offsets β for baseline electrical stimulation level (L1mV) applied to each threat avoidance parameter group-level distributional mean μ (3).

The WAIC for Model #1 was 7,237.58 with standard error (SE) = 127.64. This was used as a basis for comparison of model fit for subsequent models, with lower WAIC indicating better fit to the data. Following Model #1, we considered additional variables that might affect the subjective value computation, increasing the complexity by including one additional participant-level parameter for reward pursuit trials and the equivalent parameter for threat avoidance trials at each stage.

Model #2: SVT Model

We next incorporated a parameter to account for decreasing engagement with time on task (τ_{reward}, τ_{threat}). This parameter was tied to the (z-scored) current trial number.

The SNV for trial *t* for Model #2 was calculated as:

$$\text{SNV}_t = \gamma(\text{Stake}_t) - \kappa(\text{Effort}_t) \qquad 3(a)$$

The probability that a participant chose to engage on the trial was then given by:

$$\text{P(Engage)}_t = (1 + \exp(-[\ \text{SNV}_t - \tau \cdot \text{Trial}_t - b]))^{-1} \qquad 3(b)$$

This model provided a substantially better fit to the data over Model #1 (Model #2 WAIC=5,836.48), so the time-on-task parameter τ was carried forward to future models.

Model #3: SVTP Model

We next explored whether task performance would be better fit by allowing participants' valuation of stake to vary both linearly and nonlinearly, as suggested by utility theory. For this model we incorporated a participant-level nonlinearity parameter for stake in reward pursuit and threat avoidance trials (θ_{reward}, θ_{threat}).

We parameterized this in our model by calculating SNV at trial *t* for Model #3 as follows:

$$\text{SNV}_t = \gamma\ (\text{Stake}_t)^{\boldsymbol{\theta}} - \kappa(\text{Effort}_t) \qquad 4(a)$$

The probability that a participant chose to engage on the trial was then given by:

$$\text{P(Engage)}_t = (1 + \exp(-[\ \text{SNV}_t - \tau \cdot \text{Trial}_t - b]))^{-1} \qquad 4(b)$$

The addition of θ_{reward} and θ_{threat} improved model fit over Model #2 (Model #3 WAIC=5,741.09), so θ_{reward} and θ_{threat} were included in further models.

Model #4: SVTP SperE

Next, we evaluated a participant-level parameter that quantified the sensitivity of each participant to increases in stake per unit effort (δ_{reward}, δ_{threat}) as follows:

First, SNV at trial *t* for Model #4 was calculated as:

$$\text{SNV}_t = \gamma(\ \text{Stake}_t\ )^{\boldsymbol{\theta}} - \kappa(\text{Effort}_t) + \delta(\text{Stake}_t/\text{Effort}_t) \qquad 5(a)$$

The probability that a participant chose to engage on the trial was then given by:

$$\text{P(Engage)}_t = (1 + \exp(-[\ \text{SNV}_t - \tau \cdot \text{Trial}_t - b]))^{-1} \qquad 5(b)$$

Inclusion of δ_{reward} and δ_{threat} improved model fit over Model #3 (Model #4 WAIC=5,732.81), so the interaction term was included in further models.

Model #5: SVTP SperE Persev

We next assessed whether participants showed a tendency to perseverate in their choices (also referred to as "stickiness of choice"; η_{reward}, η_{threat}) by including a parameter to capture the repetition of the previous choice made by the participant at each trial as follows.

First, SNV at trial *t* for Model #5 was calculated as:

$$SNV_t = \gamma(Stake_t)^{\theta} - \kappa(Effort_t) + \delta(Stake_t/Effort_t) \quad 6(a)$$

The probability that a participant chose to engage on the trial was then given by:

$$P(Engage)_t = (1 + \exp(-[SNV_t - \tau \cdot Trial_t + \eta \cdot Choice_{t-1} - b]))^{-1} \quad 6(b)$$

This model improved fit to the data compared with Model #4 (Model #5 WAIC=5,717.80), so the parameter for perseveration was included in subsequent models.

Model #6: SVTP SperE Persev Psuccess

Our experimental paradigm was designed so that all levels of effort should be achievable by each individual. We calibrated each participant's highest possible required level of effort on any trial to be 85% of their maximum force demonstrated during effort calibration. However, possibly due to fatigue or fluctuation in ability or willingness to exert effort, some participants accepted some trials but still failed to achieve the level of effort required for success. We investigated whether an individual's running average within-session probability of success on trials at a given effort level influenced their choices at that effort level (ρ_{reward}, ρ_{threat}). This was parameterized in the model as follows:

First, SNV at trial *t* for Model #6 was calculated as:

$$SNV_t = \gamma(Stake_t)^{\theta} - \kappa(Effort_t) + \delta(Stake_t/Effort_t) \quad 7(a)$$

The probability that a participant chose to engage on the trial was then given by:

$$P(Engage)_t = (1 + \exp(-[SNV_t - \tau \cdot Trial_t + \rho \cdot Psuccess_{t,e} + \eta \cdot Choice_{t-1} - b]))^{-1} \quad 7(b)$$

This model provided an improved fit to the data over Model #5 (Model #6 WAIC=5,709.68), and therefore we included the parameterization of probability of success in the final model. Model #6 was the best-fitting model overall, and was used for all further hypothesis testing and inference.

Model validation

Simulated data from the generative inversion of the winning model (Model #6), see Methods, accurately reproduced participant behavior (Figures 2b, 2d and Figure S2). We confirmed that parameter values were recoverable for the winning model by simulating data using participant posterior mean parameter values and fitting the simulated data with the model. True parameter values were recovered within the 95% posterior highest density interval (HDI) of the fit parameters >=95% of the time for each parameter except for the time-on-task parameter in reward pursuit, for which 94% of the recovered posterior distributions contained the true parameter value (Figure S3). We performed a model recovery analysis (see Methods) to confirm that the winning model (Model #6) was distinguishable from other models of interest (Model #2: SVT; Model #3: SVTP; Model #4: SVTP SperE). The results are given in Figure S4. We also examined the collinearity of participant-level parameter posterior means. For the reward version of the EBDM task, there were no 2 parameters for which shared variance in values (across participants) exceeded 20%. The highest correlation observed was between the stake per effort interaction parameter and the probability of success parameter (rho = 0.43, $p < 0.01$; see Figure S7). For the aversive version of the EBDM task, the highest amount of shared variance between two parameters (across participants) was 21%, between the linear scaling of effort level parameter and the stake per effort interaction parameter (rho = 0.46, $p < 0.001$; see Figure S7.

Group-level results

Group-level distribution mean values for the baseline bias parameter, which describes baseline individual bias against trial engagement (taking into account all other specific model parameters such as stake and effort level), revealed a significantly greater bias against engagement in effortful threat avoidance than in effortful reward pursuit (b_{threat}: μ=7.09, 95% HDI=[4.65,9.83]; b_{reward}: μ=2.71, 95% HDI=[0.79,4.65]; reward – threat difference calculated across n=8,000 joint posterior distribution samples: 95% HDI = [-7.61, -1.42]). To assess the relationship between reward and threat baseline bias for individual participants, we correlated individual level posterior means for the baseline bias parameter from the reward and aversive task versions, respectively. There was a significant positive relationship between baseline bias parameter values for the two task versions across individuals (rho(48) = 0.441, p = 0.001), indicating that heightened willingness to engage in reward pursuit was also linked to heightened willingness to engage in threat avoidance, even if at a group-level participants were less willing to exert effort for the latter, (Figure 5a).

Mean parameter values for linear scaling of stake level were not significantly different between reward pursuit and threat avoidance at the group distribution level (γ_{reward}: μ=17.82, 95% HDI=[15.71,20.11]; γ_{threat}: μ=17.07, 95% HDI=[13.90,20.38]; reward – threat difference calculated across n=8,000 joint posterior samples: 95% HDI=[-2.92,4.64]). Participant level posterior mean values for the reward and aversive EBDM task versions were significantly correlated (rho(48) = 0.308, p = 0.030; Figure 5b), confirming that, in general, individuals responded similarly to changes in stake level across reward pursuit and threat avoidance.

At the group level, sensitivity to linear increases in effort was significantly higher for reward pursuit than for threat avoidance (κ_{reward}: μ=7.28, 95% HDI=[6.03,8.57]; κ_{threat}: μ=5.63, 95%

HDI=[4.51,6.84]; reward – threat difference calculated across n=8,000 joint posterior samples: 95% HDI=[0.05, 3.31]). Participant-level posterior parameter mean values for the reward and aversive EBDM task versions were significantly correlated (rho(48) = 0.425, p = 0.002; Figure 5c), indicating that the same individuals were most sensitive to increasing effort requirement across both reward pursuit and threat avoidance.

At the group level, the mean effect of the stake per effort interaction parameter was significantly greater than 0 for threat avoidance (δ_{threat}: μ=0.50, 95% HDI=[0.05,1.02]) but did not differ significantly from 0 for reward pursuit decisions (δ_{reward}: μ=0.14, 95% HDI=[-0.36,0.76]). Of note, the difference between stake per effort interaction parameter posterior means for reward pursuit versus threat avoidance did not reach significance (reward – threat difference calculated across n=8,000 joint posterior samples: 95% HDI=[-1.08,0.36]). Additionally, individual posterior mean parameter values were not significantly correlated across reward pursuit and threat avoidance, confirming a lack of consistency in contribution of the stake per effort parameter across task versions at an individual level (rho(48) = -0.098, p > 0.4; see Figure S5).

The winning model (Model #6) also included a parameter capturing nonlinearity in the subjective valuation of stake (θ). A value of $\theta = 1$ would indicate a perfectly linear subjective value for increasing stake levels. At the group level, the 95% HDI for parameter θ included 1 for both reward pursuit (θ_{reward}: μ=1.22, 95% HDI=[0.98,1.47]) and threat avoidance (θ_{threat}: μ=0.98, 95% HDI=[0.71,1.30]), indicating no significant group-level contribution of nonlinearity in stake valuation to explaining task performance. There was also no significant difference between reward pursuit and threat avoidance parameter means at the group level (reward – threat difference calculated across n=8,000 joint posterior samples: 95% HDI=[-0.14,0.61]). In addition, there was no significant relationship between individual-level posterior distribution means for reward pursuit and threat avoidance (rho(48) = 0.119, p>0.4; Figure S8).

The time-on-task parameter captured declining participant willingness to engage in effort over the course of the task. At the group level, this parameter was greater than 0 for both reward pursuit (τ_{reward}: μ=0.95, 95% HDI=[0.62,1.27]) and threat avoidance (τ_{threat}: μ=1.46, 95% HDI=[1.03,1.92]), indicating that increasing time on task was associated with participants being less willing to exert effort across both task versions. Although the group-level mean decline in engagement over time was numerically greater for threat avoidance than reward pursuit, the difference between parameter distributions did not reach significance (reward – threat difference calculated across n=8,000 joint posterior samples: 95% HDI=[-1.05,0.02]). At an individual level, there was only a trend-level relationship between posterior distribution means for reward pursuit and threat avoidance task versions (rho(48)=0.268, p=0.06; Figure 5d).

Prior probability of success for a given effort level positively influenced willingness to engage across both reward (ρ_{reward}: μ=1.58, 95% HDI=[0.51,2.66]) and threat (ρ_{threat}: μ=1.24, 95% HDI=[0.45,2.02]) task versions at the group level, with no significant difference between reward pursuit and threat avoidance (reward – threat difference calculated across n=8,000 joint posterior samples: 95% HDI=[-0.14,0.61]). There was no significant relationship between individual parameter posterior means for reward pursuit and threat avoidance (rho(48)=0.12, p=0.412).

Finally, mean prior choice perseveration was not significantly different from 0 at the group level for either reward pursuit (η_{reward}: μ= -0.14, 95% HDI=[-0.43,0.16]) or threat avoidance (η_{threat}: μ= 0.12, 95% HDI=[-0.25,0.51]), nor was the difference between reward pursuit and threat avoidance significant (reward – threat difference calculated across n=8,000 joint posterior samples: 95% HDI=[-0.72,0.18]). Additionally, there was no significant correlation between individual posterior parameter for the reward and threat versions of the task (rho(48)=-0.05, p=0.713; see Figure S8).

Supplemental Tables

Table S1: Self-report questionnaire scores for in-lab and online participants

In-Lab Participant Data (n=50)

	Mean	Std. Dev.	Range
MASQ Anxious A rousal	22	6	17 - 57
MASQ Anhedonic Depression	51	12	23 - 82
Penn State Worry Questionnaire (PSWQ)	48	15	16 - 80
State Trait Anxiety Inventory (Trait)	39	9	22 - 63
Beck Depression Inventory (BDI)	5	5	0 - 25
Apathy and Motivation Index (AMI) Behavioral Subscale	9	3	3 - 20
Temporal Experience of Pleasure Scale (TEPS) Anticipatory Subscale	45	7	29 - 59

Exploratory Factor Analysis Data (n=453)

	Mean	Std. Dev.	Range
MASQ Anhedonic Depression	25	11	17 - 83
Penn State Worry Questionnaire (PSWQ)	58	15	21 - 103
State Trait Anxiety Inventory (Trait)	49	15	16 - 80
Beck Depression Inventory (BDI)	42	11	20 - 78
Apathy and Motivation Index (AMI) Behavioral Subscale	10	9	0 - 54
Temporal Experience of Pleasure Scale (TEPS) Anticipatory Subscale	9	5	0 - 24
TEPS Anticipatory	43	9	11 - 60

Table S1: Questionnaire subscale scores for in-lab (n=50) and online exploratory factor analysis (n=453) data sets.

Table S2: Item loadings for the winning bifactor model (BF5)

Factor 1: Anxious arousal/depression

Loading	Item	Text
0.59	MASQ_25	Felt numbness or tingling in my body
0.59	MASQ_61	Felt like I was choking
0.57	MASQ_19	Felt faint
0.56	MASQ_87	Had trouble swallowing
0.53	MASQ_88	Hands were cold or sweaty
0.53	MASQ_45	Had pain in my chest
0.53	MASQ_51	Felt dizzy or lightheaded
0.51	MASQ_67	Had a very dry mouth
0.50	MASQ_73	Was afraid I was going to die
0.50	MASQ_57	Hands were shaky
0.49	MASQ_48	Had hot or cold spells
0.48	MASQ_55	Was short of breath
0.47	MASQ_79	Was trembling or shaking
0.47	MASQ_75	Heart was racing or pounding
0.45	MASQ_69	Muscles twitched or trembled
0.44	BDI_21	Lost interest in sex
0.43	BDI_5	Feel guilty
0.42	MASQ_85	Had to urinate frequently
0.41	MASQ_3	Startled easily
0.41	BDI_18	Low appetite
0.40	BDI_6	Feel I am being punished
0.39	STAI_T_11	I have disturbing thoughts
0.37	BDI_10	Crying more than usual/can't cry
0.35	BDI_14	I feel ugly/unattractive
0.34	BDI_12	Lost interest in other people
0.34	BDI_20	Worried about my health
0.32	BDI_7	Disappointed with/hate myself
0.32	MASQ_26	Felt withdrawn from other people
0.31	MASQ_66	Felt really slowed down
0.31	BDI_19	Lost weight
0.31	STAI_T_5	I feel like a failure
0.30	BDI_3	Feel like a failure
0.29	BDI_11	Irritable
0.29	MASQ_33	Felt like nothing was very enjoyable
0.29	BDI_1	Feel sad
0.29	BDI_16	Not sleeping well

0.29	STAI_T_4	I wish I could be as happy as others seem to be
0.28	BDI_17	Tired
0.26	BDI_13	Decision-making difficulty
0.26	STAI_T_20	I get in a state of tension or turmoil as I think over my recent concerns and interest
0.25	BDI_8	I blame myself
0.25	MASQ_44	Felt like there wasn't anything interesting or fun to do
0.25	MASQ_53	Felt unattractive
0.24	BDI_15	Can't get work done
0.24	BDI_2	Discouraged about future
0.23	BDI_4	As much satisfaction out of things
0.21	STAI_T_18	I take disappointments so keenly that I can't put them out of my mind
0.21	STAI_T_15	I feel inadequate
0.21	STAI_T_9	I worry too much over something that really doesn't matter
0.21	STAI_T_8	I feel that difficulties are piling up so that I cannot overcome them
0.20	STAI_T_17	Some unimportant thought runs through my mind and bothers me

Factor 2: Anhedonic depression

Loading	Item	Text
0.58	MASQ_14	Felt really happy
0.52	MASQ_23	Felt like I was having a lot of fun
0.50	MASQ_49	Was proud of myself
0.49	STAI_T_10	I am happy
0.49	MASQ_40	Felt like I had a lot to look forward to
0.49	MASQ_35	Felt like I had accomplished a lot
0.42	MASQ_86	Felt really good about myself
0.42	MASQ_58	Felt really "up" or lively
0.41	STAI_T_3	I feel satisfied with myself
0.40	MASQ_1	Felt cheerful
0.39	MASQ_78	Felt hopeful about the future
0.38	STAI_T_1	I feel pleasant
0.38	STAI_T_13	I feel secure
0.37	MASQ_30	Looked forward to things with enjoyment
0.37	MASQ_18	Felt optimistic
0.37	MASQ_72	Felt like I had a lot of energy
0.34	MASQ_36	Felt like I had a lot of interesting things to do
0.34	BDI_1	Feel sad
0.33	BDI_4	As much satisfaction out of things
0.32	MASQ_26	Felt withdrawn from other people
0.32	STAI_T_16	I am content
0.29	STAI_T_12	I lack self-confidence

0.29	STAI_T_6	I feel rested
0.28	BDI_2	Discouraged about future
0.28	STAI_T_7	I am “calm, cool, and collected”
0.27	BDI_3	Feel like a failure
0.27	MASQ_27	Seemed to move quickly and easily
0.27	MASQ_33	Felt like nothing was very enjoyable
0.24	BDI_7	Disappointed with/hate myself
0.24	STAI_T_15	I feel inadequate
0.24	BDI_14	I feel ugly/unattractive
0.22	BDI_17	Tired
0.22	BDI_12	Lost interest in other people
0.21	PSWQ_9	As soon as I finish one task, I start to worry about everything else I have to do.
0.20	MASQ_53	Felt unattractive

Factor 3: Cognitive anxiety/worry

Loading	Item	Text
0.61	PSWQ_5	I know I should not worry about things, but I just cannot help it.
0.53	PSWQ_12	I have been a worrier all my life.
0.52	PSWQ_16	I worry about projects until they are all done.
0.52	PSWQ_13	I notice that I have been worrying about things.
0.52	PSWQ_4	Many situations make me worry.
0.48	PSWQ_15	I worry all the time.
0.47	PSWQ_10	I never worry about anything.
0.44	PSWQ_3	I do not tend to worry about things.
0.43	PSWQ_14	Once I start worrying, I cannot stop.
0.43	PSWQ_8	I find it easy to dismiss worrisome thoughts.
0.43	PSWQ_6	When I am under pressure I worry a lot.
0.42	PSWQ_7	I am always worrying about something.
0.41	PSWQ_2	My worries overwhelm me.
0.37	STAI_T_9	I worry too much over something that really doesn’t matter
0.36	PSWQ_9	As soon as I finish one task, I start to worry about everything else I have to do.
0.35	STAI_T_2	I feel nervous and restless
0.34	PSWQ_1	If I do not have enough time to do everything, I do not worry about it.
0.34	PSWQ_11	When there is nothing more I can do about a concern, I do not worry about it any more.
0.34	STAI_T_20	I get in a state of tension or turmoil as I think over my recent concerns and interest
0.32	STAI_T_14	I make decisions easily
0.31	STAI_T_17	Some unimportant thought runs through my mind and bothers me
0.28	STAI_T_18	I take disappointments so keenly that I can’t put them out of my mind
0.28	AMI_5	I make decisions firmly and without hesitation.

0.25	STAI_T_12	I lack self-confidence
0.21	STAI_T_8	I feel that difficulties are piling up so that I cannot overcome them
0.21	BDI_16	Not sleeping well

Factor 4: Behavioral Apathy

Loading	Item	Text
0.61	AMI_11	I get things done when they need to be done, without requiring reminders from others.
0.53	AMI_12	When I decide to do something, I am motivated to see it through to the end.
0.53	AMI_15	When I have something I need to do, I do it straightaway so it is out of the way.
0.45	AMI_10	I don't like to laze around.
0.44	AMI_9	When I decide to do something, I am able to make an effort easily.
0.29	STAI_T_8	I feel that difficulties are piling up so that I cannot overcome them
0.28	PSWQ_2	My worries overwhelm me.
0.23	MASQ_21	Felt really bored
0.22	STAI_T_2	I feel nervous and restless
0.21	STAI_T_19	I am a steady person
0.21	BDI_15	Can't get work done
0.21	MASQ_33	Felt like nothing was very enjoyable
0.21	MASQ_39	Felt like it took extra effort to get started
0.20	MASQ_44	Felt like there wasn't anything interesting or fun to do

Factor 5 (unreversed): Anticipatory Hedonia

Loading	Item	Text
0.53	TEPS_18	When I hear about a new movie starring my favorite actor, I can't wait to see it.
0.51	TEPS_14	When I think of something tasty, like a chocolate chip cookie, I have to have one.
0.37	TEPS_15	Looking forward to a pleasurable experience is in itself pleasurable.
0.36	TEPS_11	When I'm on my way to an amusement park, I can hardly wait to ride the roller coasters.
0.36	TEPS_12	I get so excited the night before a major holiday I can hardly sleep.
0.34	TEPS_1	When something exciting is coming up in my life, I really look forward to it.
0.31	MASQ_79	Was trembling or shaking
0.30	TEPS_17	When ordering something off the menu, I imagine how good it will taste.
0.29	TEPS_16	I look forward to a lot of things in my life.
0.28	TEPS_3	When I think about eating my favorite food, I can almost taste how good it is.
0.22	BDI_14	I feel ugly/unattractive

Table S3: Model comparison (WAIC) and model key

Model comparison (WAIC)

Model	WAIC	WAIC SE
Model #1 (SV)	7,237.58	127.64
Model #2 (SVT)	5,836.48	118.87
Model #3 (SVTP)	5,741.09	118.33
Model #4 (SVTP SperE)	5,732.81	118.62
Model #5 (SVTP SperE Persev)	5,717.80	119.22
Model #6 (SVTP SperE Persev Psuccess)	5,709.68	119.71

Model key

Model	Parameters
Model #1 (SV)	b (baseline bias)<br>γ (linear stakescale)<br>κ (linear effortscale)
Model #2 (SVT)	Same as Model #1, plus<br>t (time on task)
Model #3 (SVTP)	Same as Model #2, plus<br>θ (nonlinear stakescale)
Model #4 (SVTP SperE)	Same as Model #3, plus<br>δ ($\frac{stake}{effort}$ interaction)
Model #5 (SVTP SperE Persev)	Same as Model #4, plus<br>η (repeat last choice)
Model #6 (SVTP SperE Persev Psuccess)	Same as Model #5, plus<br>ρ (previous probability of success at current effort level)

Table S3: Widely Applicable Information Criteria (WAIC, also known as Watanabe-Akaike Information Criteria) provides a measure of model fit to data in a hierarchical Bayesian framework, penalizing for number of effective parameters across the joint posterior distribution. See R-stan documentation for further detail[52].

Supplemental Figures

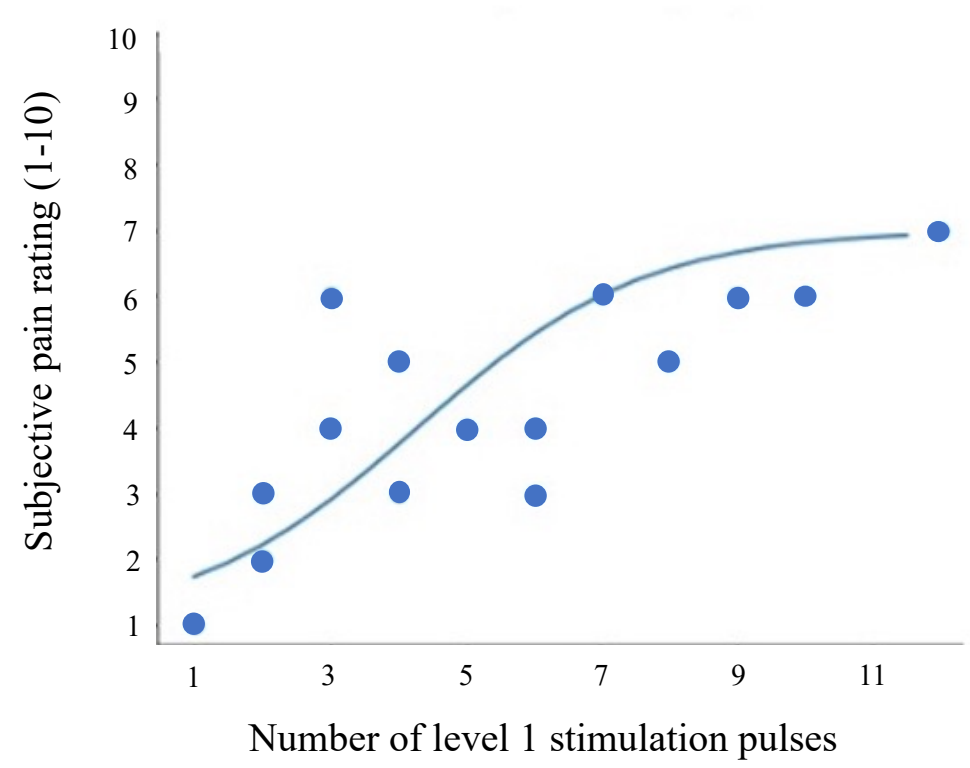

Figure S1: Illustrative sigmoidal interpolation in electrical stimulation calibration. Subjective pain rating for level #1 was calibrated to be the single pulse that a given participant selected as feeling like a small pinprick. Increasing numbers of the level #1 pulse were then delivered in train (2ms pulses spaced 12ms apart), and each participant rated the increasing pulses until their subject pain rating level #7 was reached. Ratings were then collected on an additional 14 random numbers of pulses between the participant's levels 1 and 7, and a sigmoidal curve was then fit to the relationship between pain ratings and number of stimulation pulses to interpolate between stimulation levels 2-7.

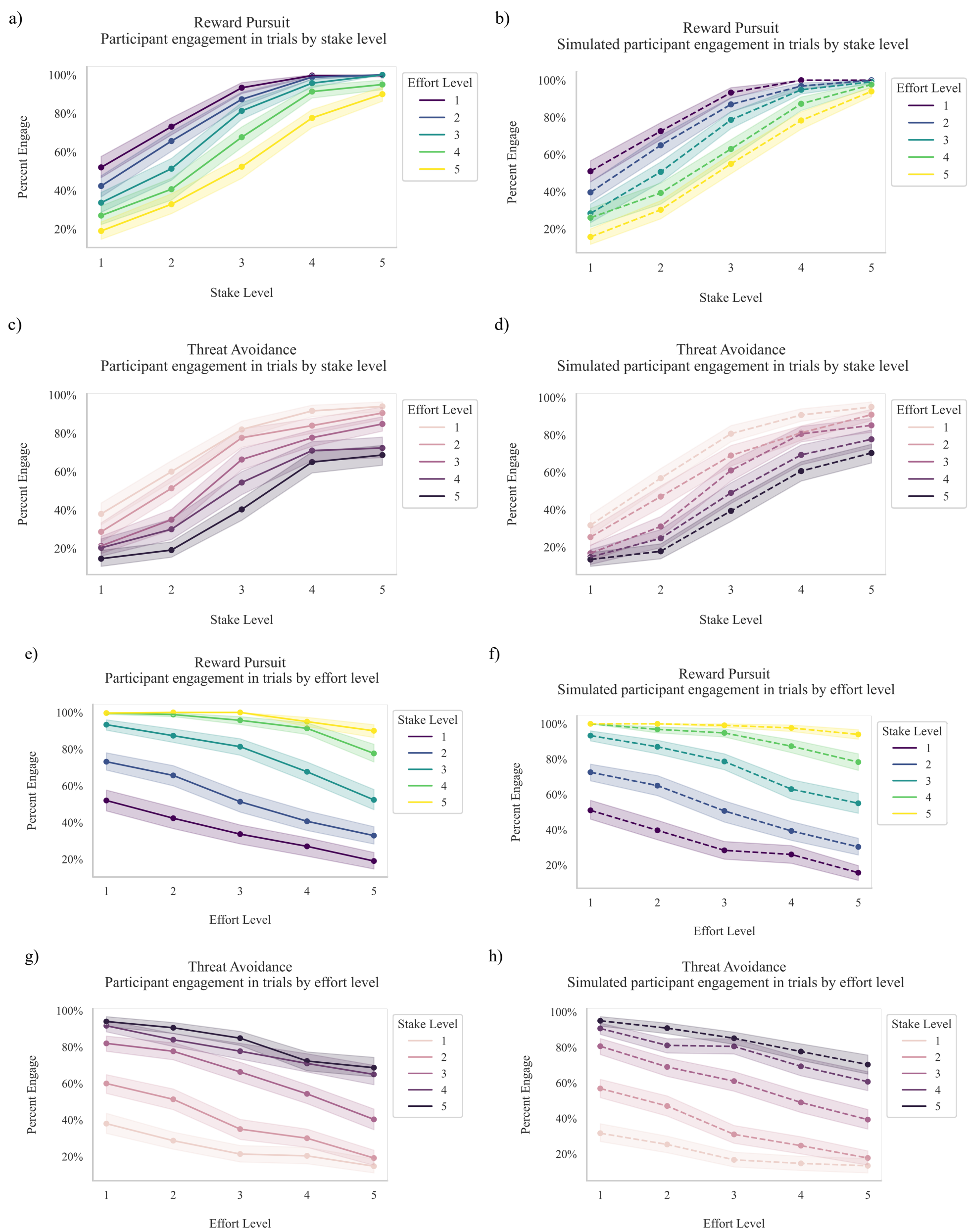

Figure S2: Participant choice data and choice data simulated from winning model (Model #6); 2-dimensional views. Panel a) Participant data: percentage of trials for which participants chose to engage (i.e. to exert effort; y-axis) in reward pursuit EBDM task as a function of stake level (x-axis) and required level of effort (as indicated by data point and line color, see legend). Point indicates mean engagement across participants at given effort and stake level, and shaded line shows regression and 95% CI for each effort level across stake levels. Panel b) Analogous plot to panel a, but using data simulated by the winning model (Model #6) for reward pursuit. Panel c) Participant data: percentage of trials where participants chose to engage (i.e. to exert effort; y-axis) in threat avoidance EBDM task as a function of stake level (x axis) and required level of effort (as indicated by data point and line color, see legend). Point indicates mean engagement across participants at given effort and stake level, and shaded line shows regression and 95% CI for each effort level across stake levels. Panel d) Analogous plot to panel c, but using data simulated by the winning model (Model #6) for threat avoidance. Panels e) – h): Analogous to panels a) – d), but with effort level on x-axis and stake level indicated by data point and line colors (see legend). Point indicates mean engagement across participants at given stake and effort level, and shaded line shows regression and 95% CI for each stake level across effort levels.

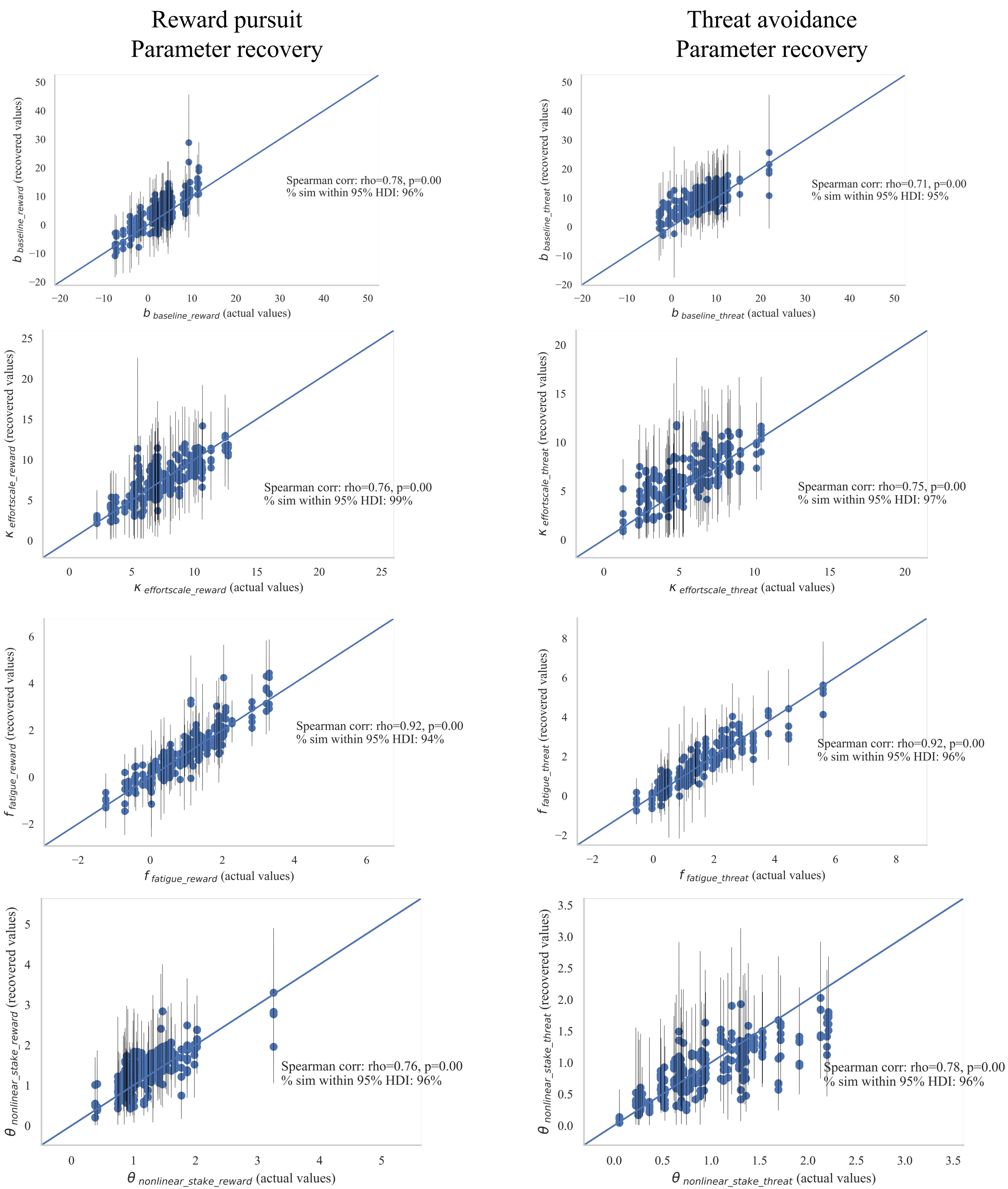

(continued on next page)

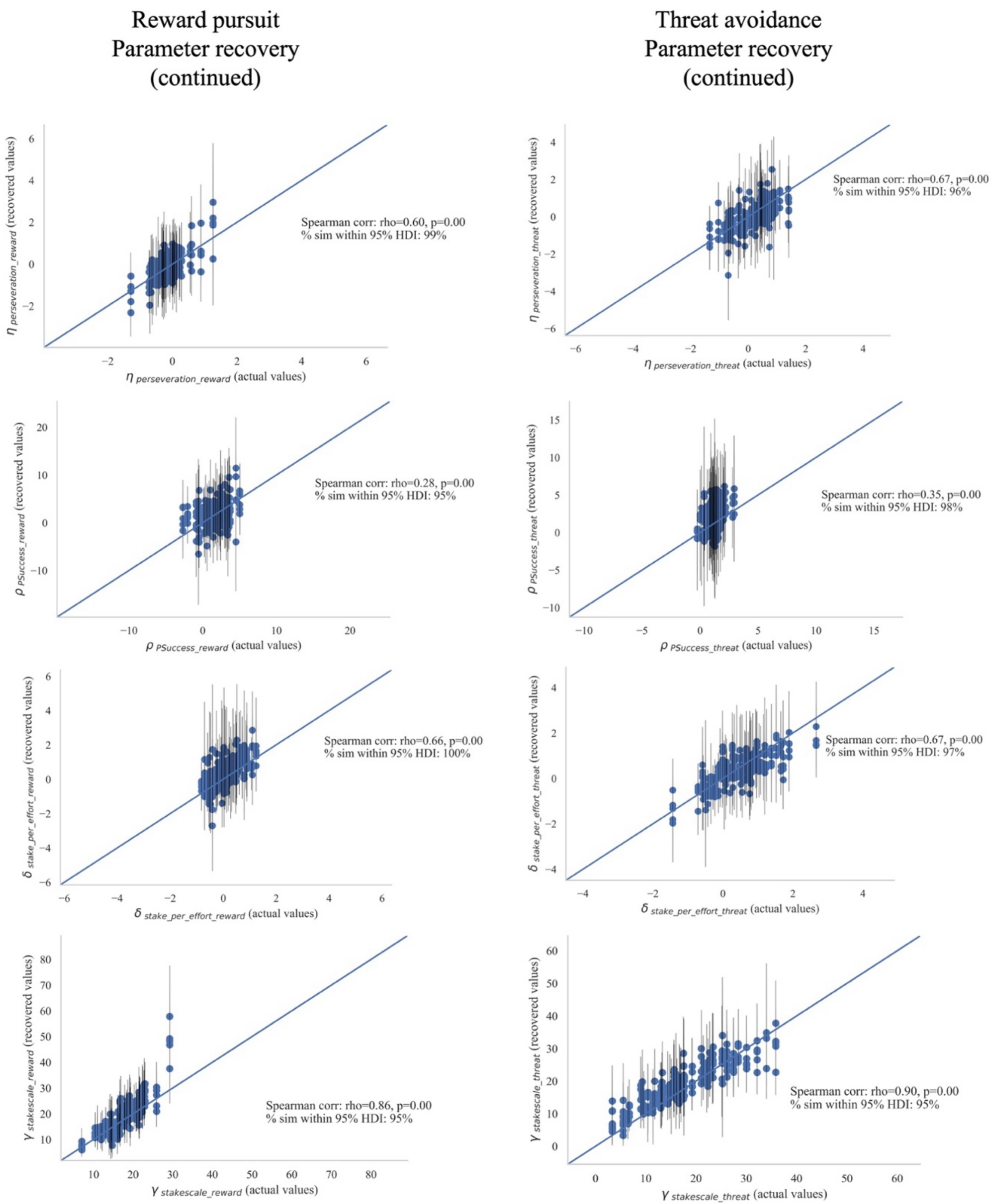

Figure S3: Parameter recovery for winning model (Model #6). Four data sets of n=50 participants each were simulated using Model #6 participant-level posterior parameter mean values. Simulated data sets were fit using Model #6. Each plot shows recovery for one parameter; left column shows reward pursuit parameters, right column shows threat avoidance parameters. For each plot: x-axis shows parameter values used to generate simulated data; y-axis shows the posterior mean (point) and 95% highest density interval (HDI; line) of recovered parameter values. Spearman correlations are shown for actual parameter values with posterior means of recovered parameters across simulated data (n=200). The percentage of actual parameter values within the 95% posterior HDI of the recovered parameters is shown in text for each parameter; 95% or higher indicates strong parameter recovery in a hierarchical Bayesian framework.

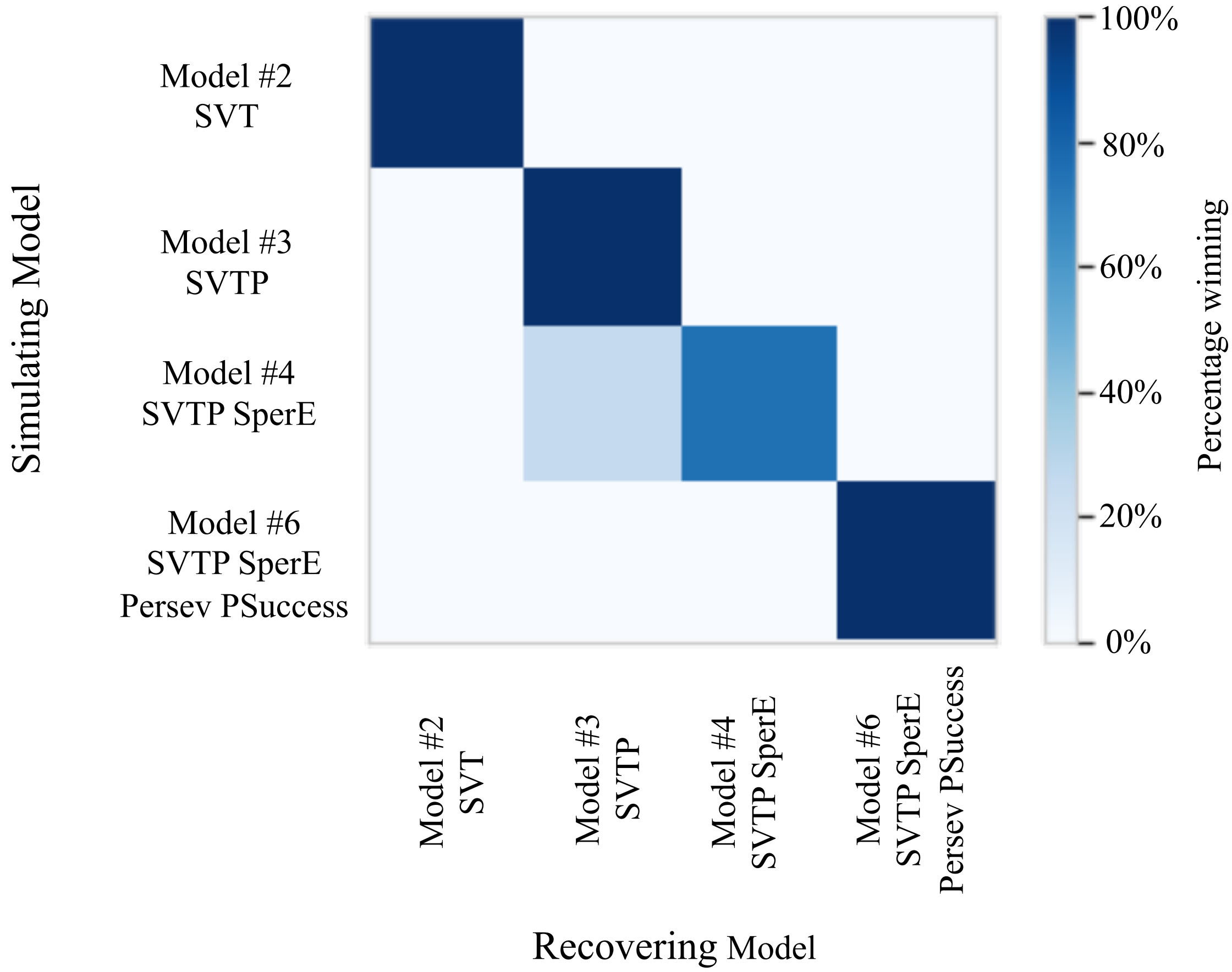

Figure S4: Model recovery. Four data sets of n=50 participants each were simulated for each model shown using the actual participant posterior parameter means from that model. Each data set was then fit by all four models, and the winning model was selected as the model with the lowest WAIC. For each simulating model (y-axis) by each recovering model (x-axis), the plot shows the percentage of occasions on which the model provided the best fit to the data.

a)

Factor	Interpretation	R^2
G	Internalizing	0.721
F1	Mixed anxiety and depression (somatic)	0.855
F2	Anhedonic depression	0.797
F3	Worry	0.648
F4	Apathy (behavioral)	0.898
F5_rev	Anticipatory anhedonia	0.841

b)

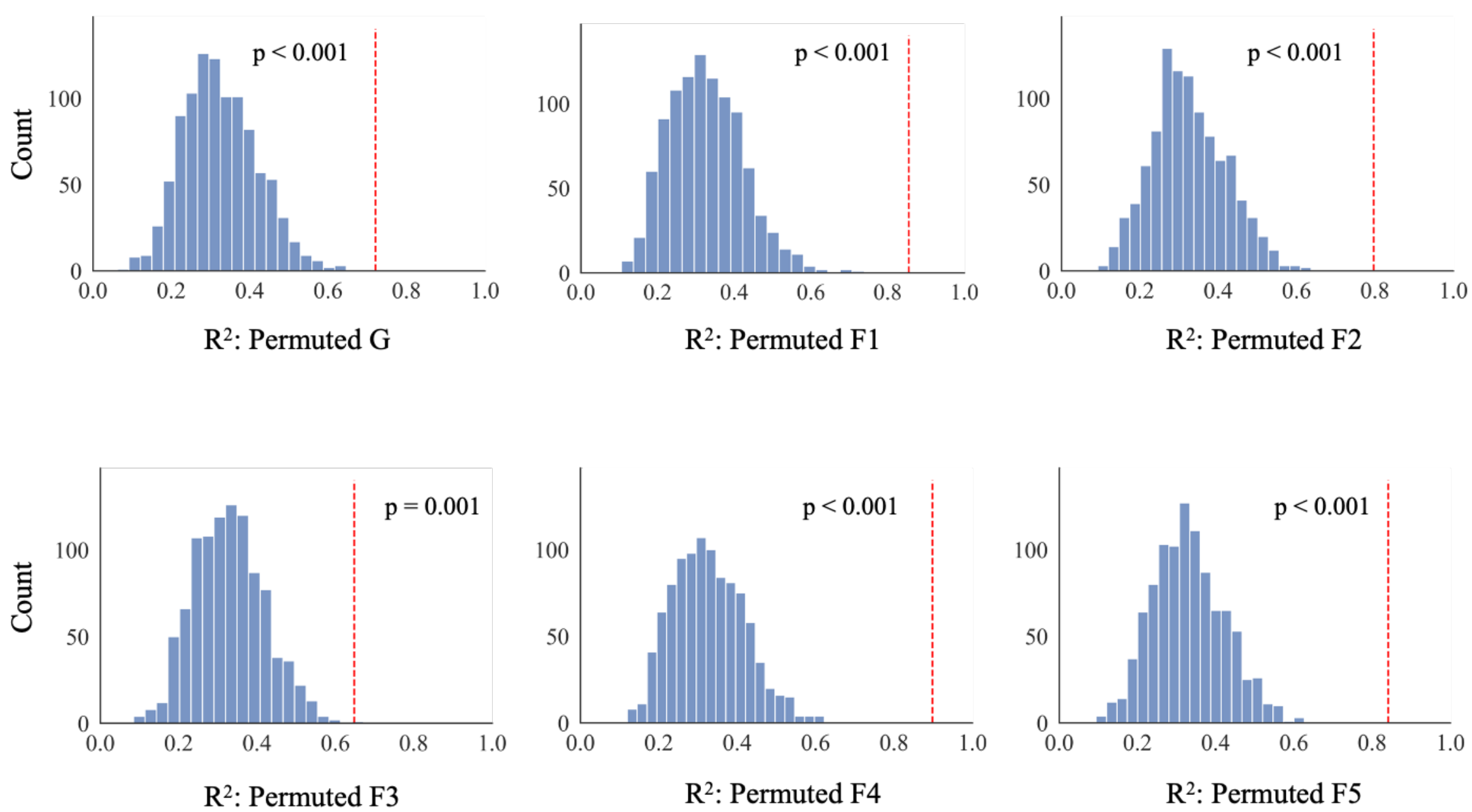

Figure S5. Explanatory value of model in predicting individual trait affect. a) Amount of variance (R^2) in latent factor scores explained by behavioral model parameters. Linear regression was performed for each latent factor score using reward pursuit and threat avoidance model parameters from the winning model (Model #6) as predictors. Baseline parameter values prior to adjustment for parameter effects of latent trait scores were used. b) Permutation testing of predictive significance. For each latent factor, n=1000 permutations were run predicting randomly permuted scores by baseline model parameters (R^2 results for permutations shown as histograms; actual predictive result from a) shown as dashed red line). Significance of actual predictive values were calculated using the percentage of permutation samples exceeding the actual predictive value.

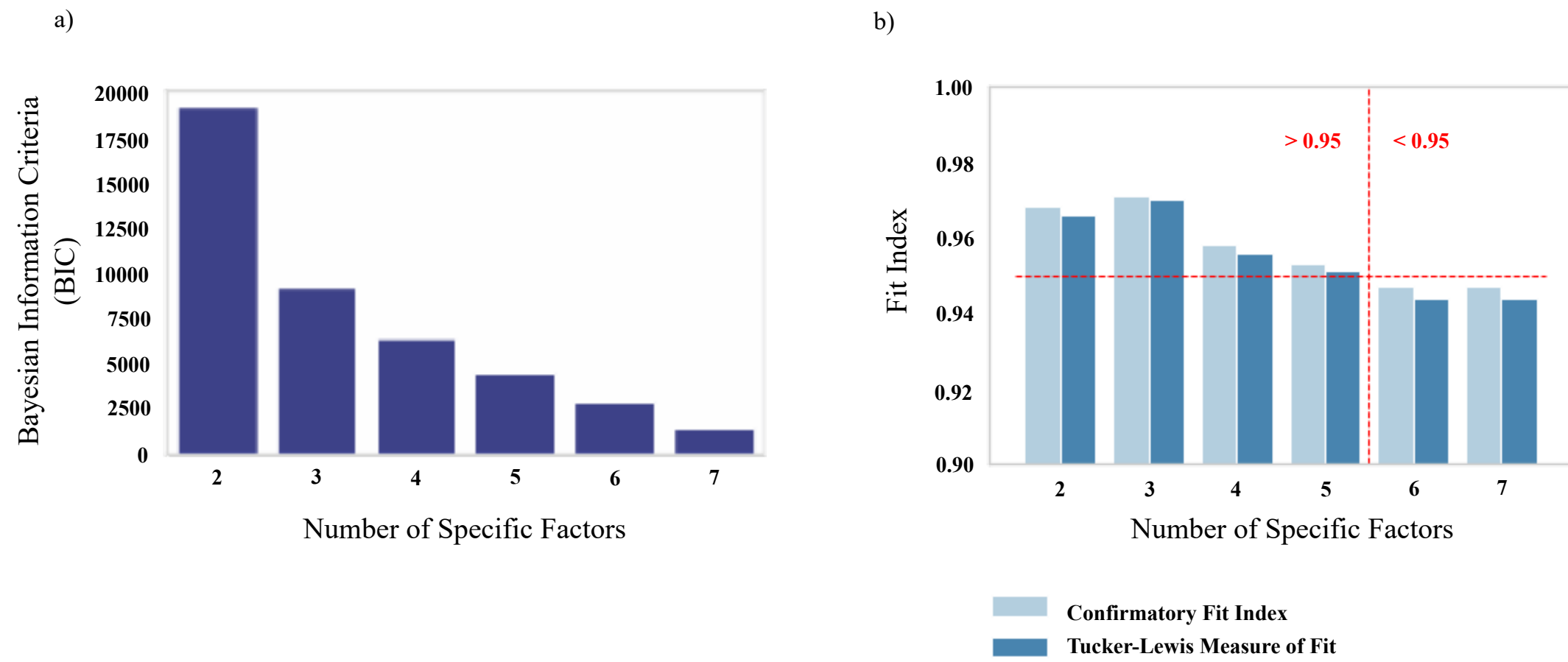

Figure S6. Comparison of fit indices between bifactor models of latent subdimensions. a) Bifactor models with 1 to 7 specific factors were fit to item-level questionnaire responses using the exploratory dataset. The Bayesian Information Criterion (BIC, arbitrary units) for each model is presented; this reveals that model fit improves as the number of specific factors increases. b) The comparative fit of each bifactor model was further assessed using the confirmatory dataset via both the confirmatory fit index (CFI) and the Tucker-Lewis measure of fit. This revealed a highly credible fit (> 0.95) for models with five or fewer specific factors. Consideration of both exploratory and confirmatory model fit indices led us to select the bifactor model with five specific factors.

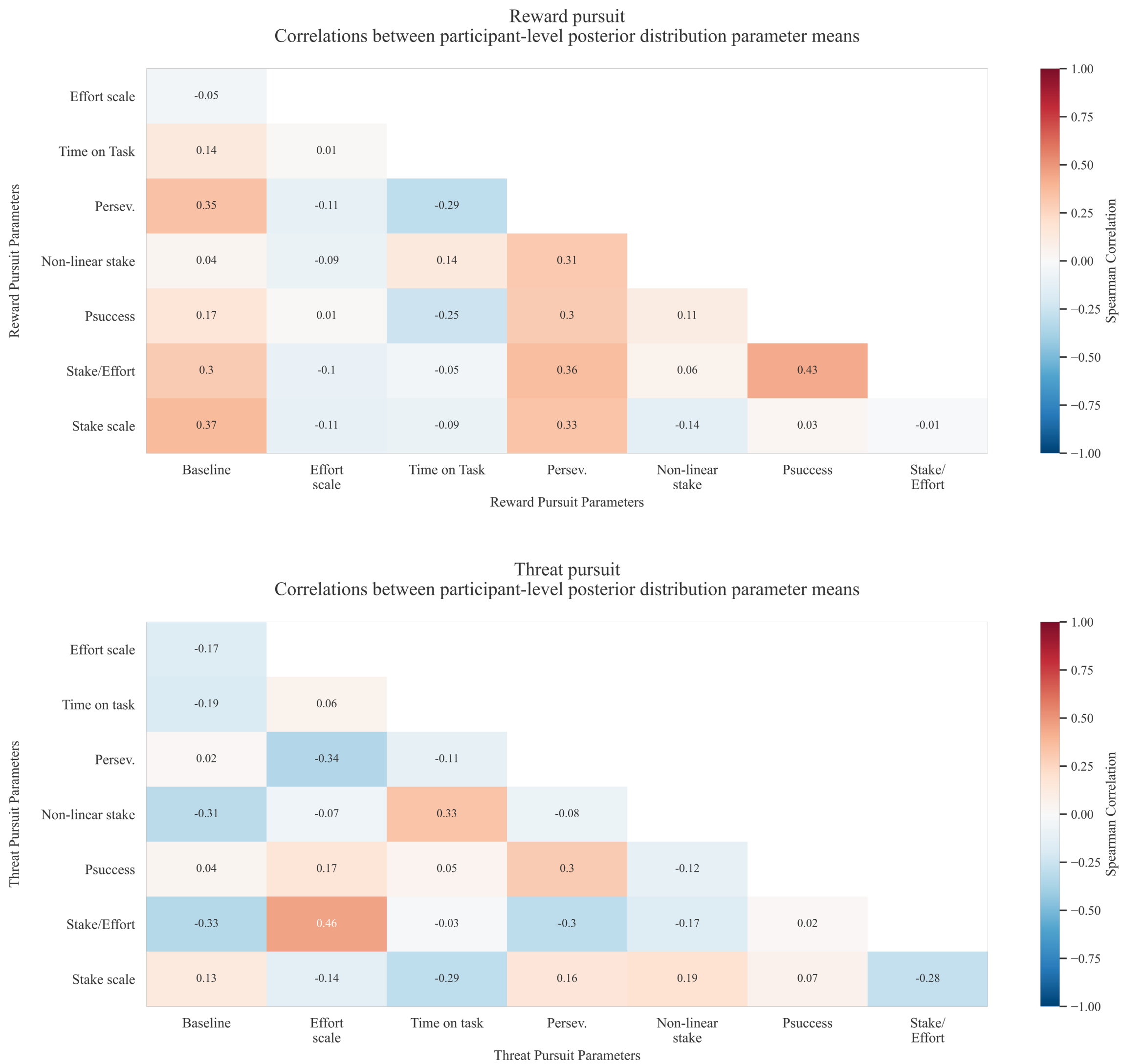

Figure S7: Correlations between participant-level parameter posterior means. Top panel: Spearman correlations between participant-level parameter posterior means across participants (n=50) are presented for reward pursuit parameter values estimated by the winning model (Model #6). Bottom panel. Spearman correlations between participant-level parameter posterior means across participants (n=50) are presented for threat avoidance parameter values estimated by the winning model (Model #6).

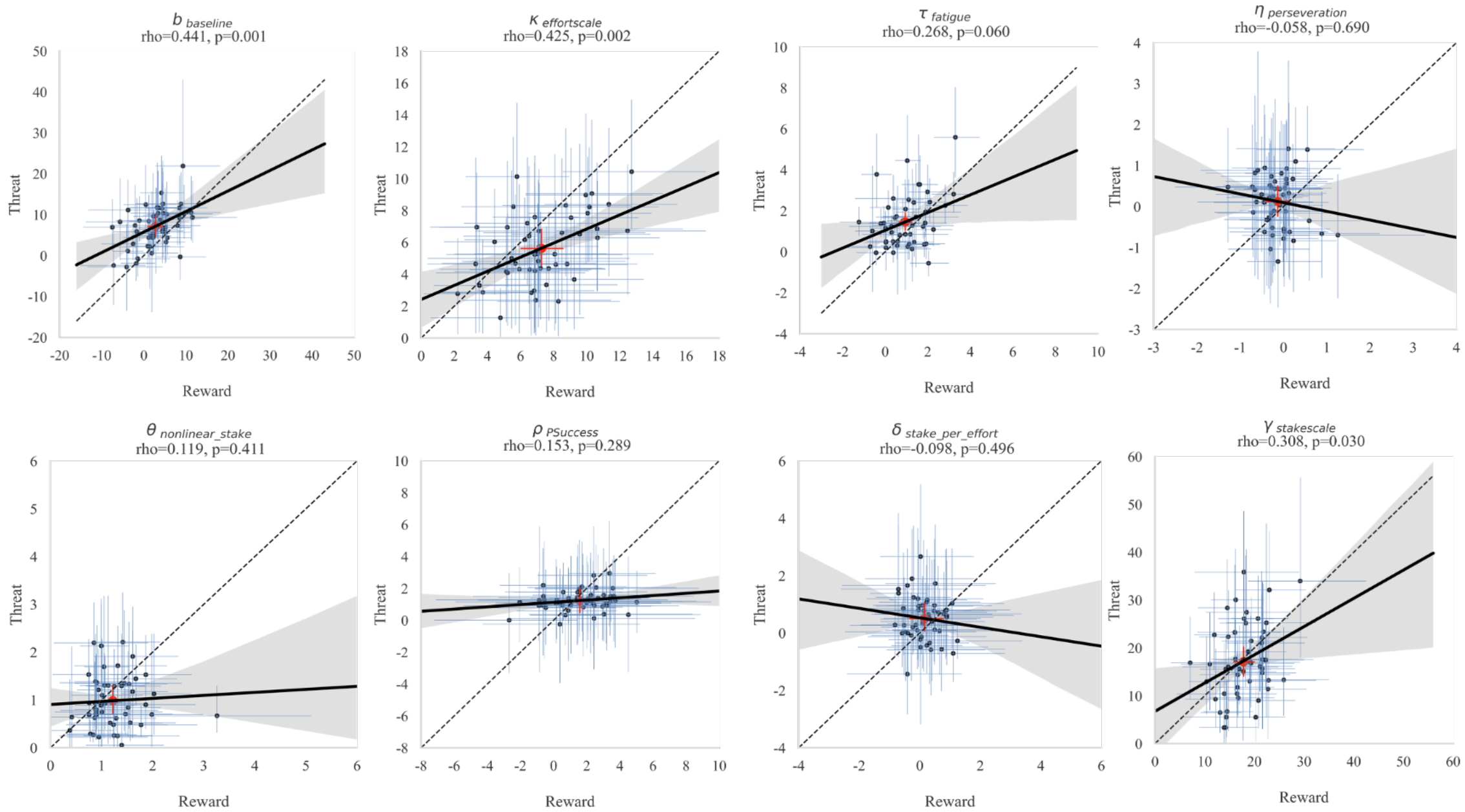

Figure S8: Participant parameters for reward pursuit and threat avoidance versions of the EBDM task. Each plot shows participant parameter posterior means and 95% HDIs for reward pursuit (x-axis) versus threat avoidance (y-axis) versions of EBDM task for the parameter specified. The solid black line shows the best fit linear regression line, with the shaded region showing the associated 95% confidence interval; the dashed line shows unity (threat and reward parameter posterior means are of equal value). The group-level distribution mean and 95% HDI are shown in red for each parameter.

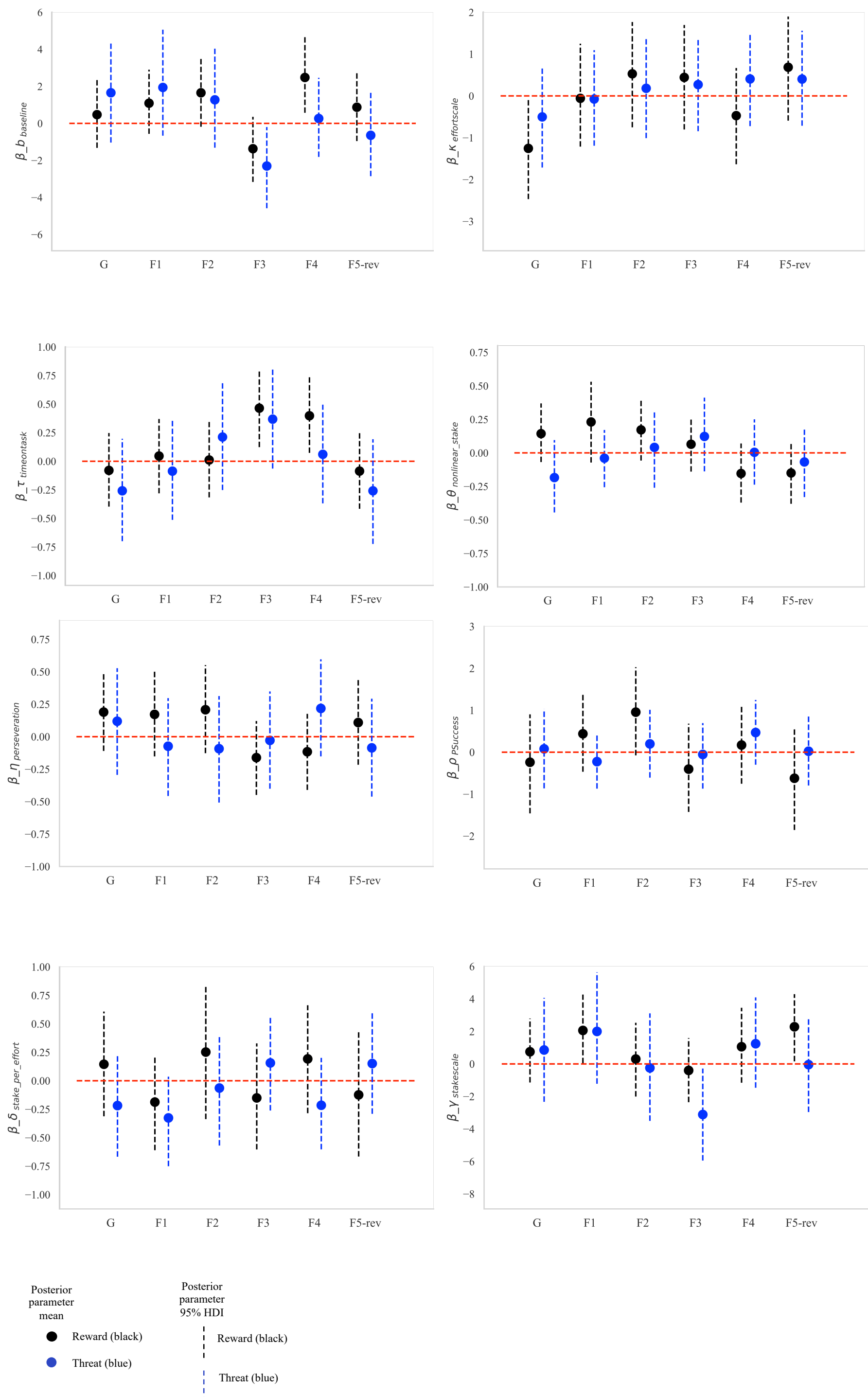

Figure S9: Effects of latent factors of negative affect on each parameter of the winning model (Model #6). Each plot shows posterior mean (solid dot) and 95% highest density interval (HDI; dashed line) for reward pursuit (black) and threat avoidance (blue) β parameters (with parameter specified on y-axis). Each β parameter describes the offset to the corresponding parameter group mean due to participant scores for each of the latent factors from the winning bifactor model as specified on the x-axis.

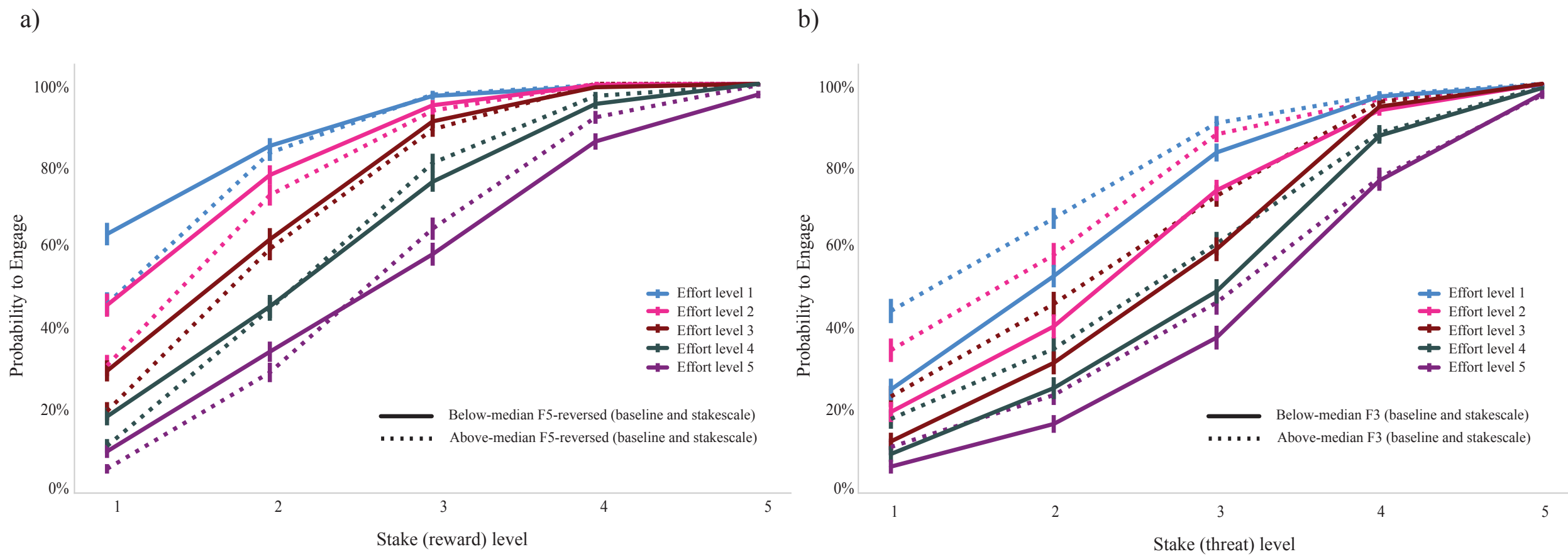

Figure S10: Latent factors capturing anticipatory anhedonia (F5-reversed) and worry (F3) show stake-level dependent influences upon effortful pursuit of reward and avoidance of threat, respectively. Each plot shows simulated participant data, with stake level on the x-axis, probability of trial engagement (decision to exert effort) on the y-axis, and data for each effort level plotted separately. Panel (a) shows simulated reward pursuit data using the posterior mean of participants' values for each parameter except for baseline bias (b) and linear stakescale (γ) parameters which were adjusted to reflect the mean across participants with above-median (dotted line) or below-median (solid line) scores on F5_reversed. Panel (b) shows an equivalent simulation for threat avoidance data with baseline bias (b) and linear stakescale (γ) parameters adjusted to reflect the mean across participants with above-median (dotted line) or below-median (solid line) scores on F3. We adjusted the baseline bias parameter, in addition to the stake parameter, given the significant relationship between F3 scores and baseline bias and to better capture the effect of the stake parameter with the baseline bias parameter included in the model. The simulations reveal that both anticipatory anhedonia (above median F5-reversed scores) and worry (above median F3 scores) primarily impact trial engagement at low stake levels, with anticipatory anhedonia being linked to reduced willingness to exert effort in pursuit of reward at low stake levels and worry to increased willingness to exert effort in avoidance of threat at low stake levels.
